# A Model of Pattern Separation by Single Neurons

**DOI:** 10.3389/fncom.2022.858353

**Published:** 2022-04-29

**Authors:** Hubert Löffler, Daya Shankar Gupta

**Affiliations:** ^1^Independent Scholar, Bregenz, Austria; ^2^College of Science and Humanities, Camden County College, Husson University, Bangor, ME, United States; ^3^Department of Biology, Camden County College, Blackwood, NJ, United States

**Keywords:** synfire chain, subthreshold membrane potential oscillations, temporal coding, expansion recoding, pattern separation

## Abstract

For efficient processing, spatiotemporal spike patterns representing similar input must be able to transform into a less similar output. A new computational model with physiologically plausible parameters shows how the neuronal process referred to as “pattern separation” can be very well achieved by single neurons if the temporal qualities of the output patterns are considered. Spike patterns generated by a varying number of neurons firing with fixed different frequencies within a gamma range are used as input. The temporal and spatial summation of dendritic input combined with theta-oscillating excitability in the output neuron by subthreshold membrane potential oscillations (SMOs) lead to high temporal separation by different delays of output spikes of similar input patterns. A Winner Takes All (WTA) mechanism with backward inhibition suffices to transform the spatial overlap of input patterns to much less temporal overlap of the output patterns. The conversion of spatial patterns input into an output with differently delayed spikes enables high separation effects. Incomplete random connectivity spreads the times up to the first spike across a spatially expanded ensemble of output neurons. With the expansion, random connectivity becomes the spatial distribution mechanism of temporal features. Additionally, a “synfire chain” circuit is proposed to reconvert temporal differences into spatial ones.

## Introduction

A key task navigating the world is to distinguish between similar but different objects, places, or contexts. The neuro-computational function thought to underlie this ability is referred to as “pattern separation.” It is defined as the process by which similar neuronal input is converted into a less similar output that represents the input. Indeed sometimes the pattern separation gained popularity as a research topic (Drew, 2010; Leal and Yassa, 2018), however, up to date it has been studied almost exclusively on the bases of spatial patterns. Many functional and structural neural circuit mechanisms have been found, each of which contributes to the spatial pattern separation. Expansion recoding, sparse synaptic connectivity to the output neurons, and Winner Takes All (WTA) competition through inhibition have so far mainly been considered as relevant mechanisms for spatial pattern separation: Marr (1971), O’Reilly and McClelland (1994), Chistiakova and Volgushev (2009), Aimone et al. (2011), Yassa and Stark (2012), Rolls (2012, 2013, 2016), Cayco-Gajic and Silver (2019), and Sakon and Suzuki (2019) as review.

### Spatial or/and Temporal Separation

Given the spatiotemporal characteristics of most physical stimuli, sensory information is encoded both spatially and temporally. Temporal precision seems to be critical for the accurate representation of physical sensory stimuli, which at least is shown by spike-timing-dependent plasticity (STDP). Therefore, we now focus on differences in the temporal properties of processed patterns, especially on the delays of neuronal answers to input patterns as a kind of temporal coding.

The explicit application of temporal coding to the models of pattern separation has rarely been done, although much evidence has been reported that temporal coding is useful for pattern separation. The local properties of activated neurons (spatial coding) or frequency changes (rate coding) up to date are mainly used as criteria for the separation of patterns.

In fact, there is growing evidence for temporal encoding strategies in neural networks. Indeed, there is an increasing evidence of their direct involvement in separation processes. Thus, Lisman and Jensen (2013) directly relate to the pattern separation by spiking breaks produced by gamma frequency arrest. They assume that different spatial information is represented in different gamma subcycles of a theta cycle.

Pernía-Andrade and Jonas (2014) suggest that rate coding schemes in dentate gyrus granule cells, which are ascribed to pattern separation, would be inadequate compared with temporal coding schemes. Their results are consistent with the idea that a temporal coding scheme is used in dentate gyrus granule cells.

Madar et al. (2019) provided the first experimental evidence of temporal coding strategies for pattern separation in the hippocampus. Examining the temporal properties of input and output patterns rather than differences in firing rates or firing locations, the authors showed that the suprathreshold responses of some gyrus cells were highly decorrelated compared with their inputs. The authors presented different ways to measure the similarity between spike trains and suggested that pattern separation could be achieved by multiplexed neural codes.

Our model uses spike patterns as input, generated by different numbers of neurons firing at different high gamma frequencies. Input similarity is measured by spatial overlap. As an output, we measure both the temporal and spatial properties of patterns in the output. We propose that the separation is primarily made by time to first output spike. This temporal property arises in each individual output-neuron and is used as the basis for the separation processes. Only in a further step is the temporal code extended by a spatial code through expansion recoding or by synfire chain.

In essence, our model shows that temporal encoding by time-delays to the first spike allows the individual output neurons to differentiate their input, especially when this process is assisted by rising subthreshold membrane potential oscillations (SMOs). We propose that the first step in separating spatially overlapping input patterns is achieved through temporal encoding: depending on the spatiotemporal input and the theta phase of SMOs, output neurons will fire sooner or later. Only through a second mechanism, the temporal properties of activated neurons extended by a spatial code, namely, through expansion recoding and sparse connectivity in combination with a competing inhibition process.

As an additional option for the interaction between temporal and spatial properties of patterns, a neural mechanism is used to convert temporal differences into spatial ones using a simple synfire chain model (Abeles, 1982; Ikegaya et al., 2004; Hosaka et al., 2008; Larson et al., 2010). Such models suggest a succession of end-to-end excitatory neurons (neuronal chain). An input spike propagates along the chain of neurons with synaptic delays.

### Separation by Dentate Gyrus

Spatial pattern separation was ascribed to the dentate gyrus of the hippocampus (Berron et al., 2016). The study about temporal pattern separation from Madar et al. (2019) also examined how the input-output transformation of multiple hippocampal cell types works in the terms of pattern separation. The assignment of pattern separation to the dentate gyrus should also apply to temporal properties. The results of Pernía-Andrade and Jonas (2014) agree with this idea because dentate granule cells use a temporal coding scheme. Braganza et al. (2020) point out that in addition to the feedback, inhibition sparsity and temporal oscillations in the dentate gyrus have a critical influence on a proposed pattern separation function. However, it should be noted that pattern separation is not unique to this area of the brain. Similar phenomena also manifest in other neural circuits, e.g., the cerebellar cortex and insect mushroom body. However, recent studies from human single-neuron recordings have suggested that there may be no pattern separation in the human hippocampus (Quiroga, 2020). Others insist that experimental evidence does not rule out pattern separation in the hippocampus (Rolls, 2021; Suthana et al., 2021).

## Materials and Methods

### Model

**FIGURE 1:**
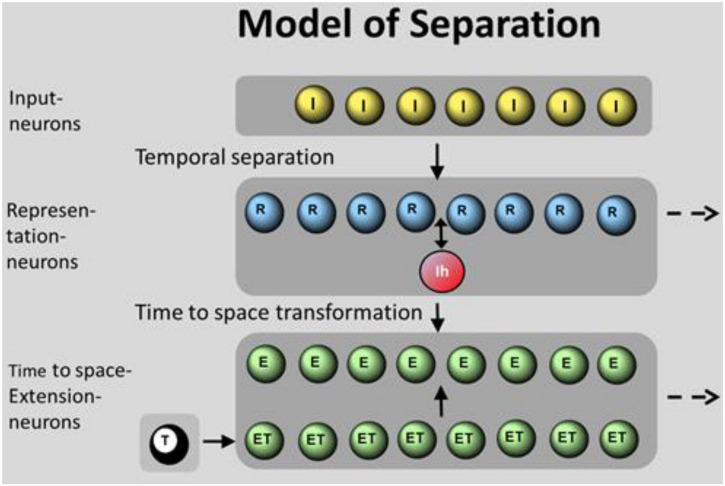
Model of spatiotemporal separation with 3 neuronal ensembles: I-neurons (I), R-neurons (R), and E-neurons (E). In total, 8 I-neurons are randomly connected to a varied number of R-neurons generating separation (Figure 8). R-neurons are fully connected to the global inhibitory neuron Ih that is reconnected to all R-neurons. R-neurons are fully connected to E-neurons, generating time to space transformation by the help of ET-neurons as “synfire wave.” The activation of ET-neurons is initiated by the T-neuron (T). The activation of the first ET-neuron (ET) is propagated to the neighboring ET-neuron. Spiking ET-neurons activate the parallel E-neurons where times of excitatory postsynaptic potentials (EPSPs) “travel” from one neuron to the next.

A spiking neural network (SNN) model is presented to demonstrate a prototypical approach of pattern separation. It consists of 3 neuronal ensembles: the input neurons (I-neurons), the representation neurons (R-neurons), and the time to space extension neurons (E-neurons), each accomplishing a specific task (Figure 1). Beside the input function of the ensemble of I-neurons (as shown in section “Summation in R-Neurons”), the other two ensembles solve the following tasks: spatial and temporal separation as key part of the ensemble of R-neurons and time to space transformation as an additional option of the ensemble of E-neurons. Separation is generated between I-neurons and R-neurons, extension from time to space (temporal parameters are complemented by spatial parameters) is generated between R-neurons and E-neurons.

The random connectivity between I-neurons and R-neurons is organized as follows: eight I-neurons have excitatory synapses with every six dendrites of 1–128 R-neurons, representing an expansion factor (EF) of 0.125–16. Each of the dendritic branches of R-neurons is connected by 3 synapses from I-neurons. The connections of the single dendritic branches are diluted, i.e., they receive input only from 3 to 6 I-neurons, not of all. For example, as shown in Figure 8.

**FIGURE 2:**
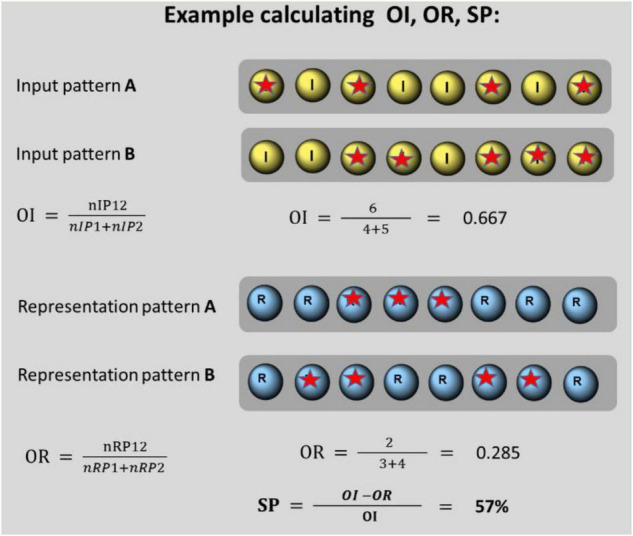
Spatial overlap of input patterns (OI), output patterns (OR), and separation power (SP) calculated for a pair of 8 I-neurons: pair A consists of 4 spiking neurons (designated by a red star) and pair B consists of 5 spiking neurons. In total, 3 pairs (6 neurons) fire together. OI amounts to 0.667. The corresponding representation pair contains one time 3 and one time 4 spiking neurons. One pair (2 neurons) spike together. OR amounts to 0.285. This leads to a separation power of SP = 57%.

**FIGURE 3:**
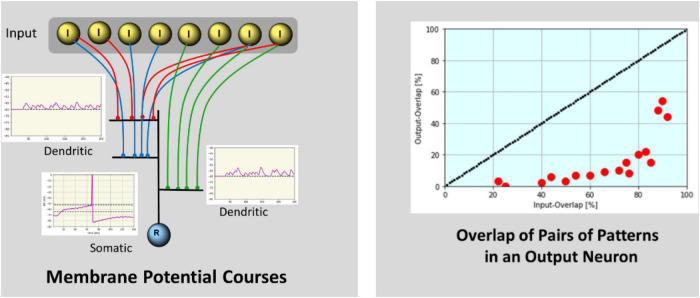
**Left:** Membrane-potential courses by simulation. Courses of two dendritic branches each connected with 4 (red and green) I-neurons are shown. Each I-neuron fires with a specific frequency resulting in subliminally summed EPSPs at the single branch. The somatic potential of the R-neuron is a combination of the summed input from the dendrites and the somatic SMO (5 Hz), resulting in a ramping activity course. The neuron will spike when increasing membrane potential reaches the spiking threshold. After spiking an inhibitory feedback potential reduces the membrane potential to prevent further spiking. **Right:** The percentage of overlap of pairs of output patterns is shown in relation to the percentage of overlap of the corresponding pairs of input patterns. Separation is demonstrated by values below the diagonal marking zero separation.

**FIGURE 4:**
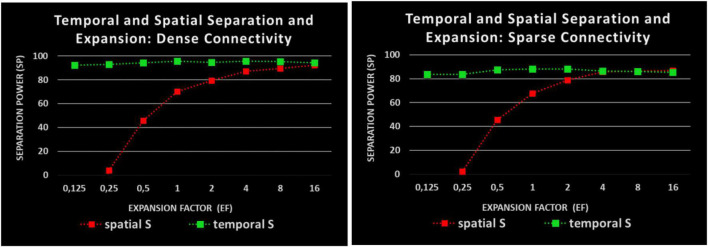
Impact of EF to SP: two kinds of separation measurement: red: spatial separation; green: temporal separation; Abscissa: EF; Ordinate: SP as a reduction of overlap. **Left:** Dense connectivity, **Right:** Sparse connectivity. Sparse connectivity reduces the temporal but not the spatial SP.

**FIGURE 5:**
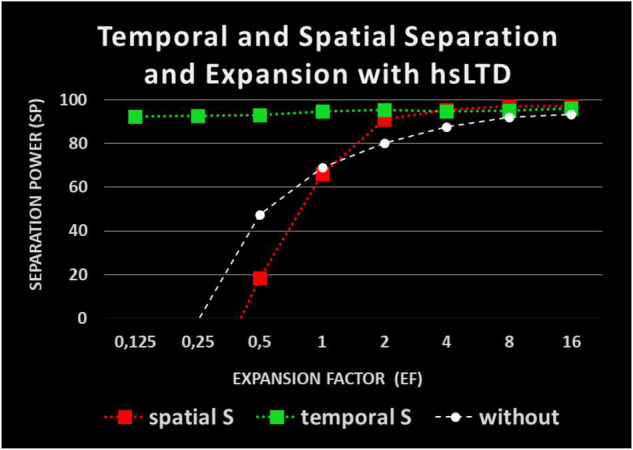
Impact of heterosynaptic long-time depression (hsLTD) on pattern separation: red: spatial separation with hsLTD; green: temporal separation with hsLTD; white: spatial separation without hsLTD.

**FIGURE 6:**
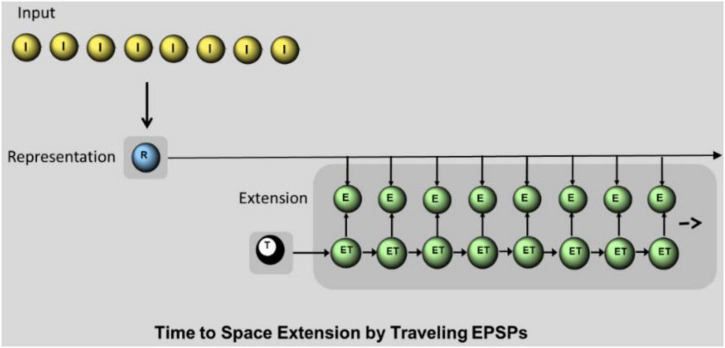
Time to space extension mechanism by synfire chain. I: I-neurons sending spatiotemporal pattern to the dendrites of R-neurons. R: single R-neuron representing the input by temporally precise spike generation. E: E- neurons. Each E-neuron receives the input from the R-neuron and an additional input from the parallel ET-neuron. Only the simultaneous input from R and ET leads to a spike in E-neuron. T: T-neuron, initiates sequential spikes in ET-neurons.

**FIGURE 7:**
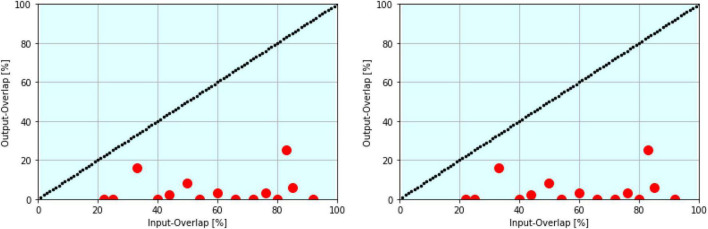
Example of temporal separation in R-neurons (left) and spatial separation in E-neurons (right). Output overlap (*y*-axis) vs. input pattern overlap (*x*-axis) is shown. In both cases SP = 91.3%.

**FIGURE 8:**
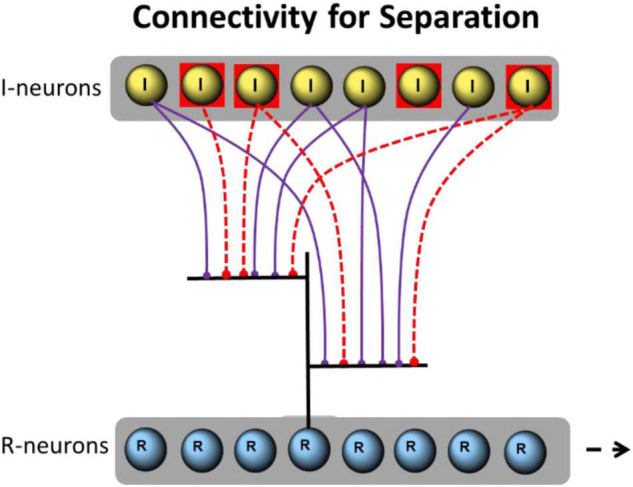
Example of implemented connections between I- and R-neurons. A single R-neuron with 6 dendritic branches (only two are shown) is excitatory connected through 6 I-neurons (violet). A total of 4 I-neurons are activated (red squares). Their spikes are propagated to the R-neuron (red dotted connections).

For simulations, 3–6 of 8 I-neurons propagate high-gamma aligned spike trains to the 6 dendritic branches of 1–128 R-neurons. A total of 3 synapses are formed per branch. This results in 18 synapses per R-neuron from 8 randomly selected I-neurons. So first, we are using a **dense** connectivity of average 2.25 connections per I-neuron. Additionally, we investigate the separation effects if **sparse** connectivity is applied. For sparse connectivity, 3–6 of 8 I-neurons propagate the spike trains to the 6 dendritic branches of R-neurons but only one synapse is formed per branch. This results in only 6 synapses per R-neuron form 8 randomly selected I-neurons, a sparse connectivity of average 0.75 connections per I-neuron a third of the former dense connectivity.

### Neural Mechanisms Underlying Separation

#### Definition of Overlap

Largely, pattern separation is defined as a putative process that the brain organizes to transform similar inputs into less similar outputs (e.g., Sakon and Suzuki, 2019). Separation is performed as a reduction of overlap from input and output patterns. An example is shown in Figure 2. This definition up to date relates mainly to the spatial dimension of separation. We extend the definition of the output overlap to the temporal dimension. The input overlap still remains defined by the spatial dimension.

After the presentation of input patterns, their spatial overlap (OI) is compared with the spatial or temporal overlap of output patterns (OR).


(1) $$\text{OI}=\,\frac{\text{nIP}\,12}{n\,I\,P\,1+n\,I\,P\,2}$$

*Where, OI is the similarity of a pair of input patterns calculated by their spatial overlap. nIP12 is the number of activated I-neurons present in both matched patterns, nIP1 is the number of activated neurons in input pattern 1, and nIP2 is the number of activated neurons in input pattern 2.*

The similarity of output patterns is calculated by the relation of the number of spiking neurons present in both matched patterns to the total number of spiking representation neurons. Spatial and temporal overlap is calculated separately.


(2) $$\text{OR}=\,\frac{n\,R\,P\,12}{n\,R\,P\,1+n\,R\,P\,2}$$

*Where, OR is the similarity of a pair of output patterns calculated by their overlap. nRP12 is the number of spiking R-neurons existing in both patterns, nP1is the number of spiking R-neurons in output pattern 1, and nRP2 is the number of spiking R-neurons in output pattern 2. If spatial separation is measured, similarity is defined by the amount of identical locations of emerging spikes. If temporal separation is measured, similarity is defined by the number of synchronously spiking output neurons.*

The OI of all possible pairs of input patterns (without equal ones) and OR of all possible pairs of corresponding output patterns are matched. The separation power (SP) is calculated by the percentage of reduction of overlap of all partially or totally different input patterns.


(3) $$\text{SP}=\,\frac{O\,I-O\,R}{O\,I}$$

*Where, SP is the separation power during designated conditions. It computes the reduction of similarity in the output patterns when compared with the input patterns. OI is the averaged overlap of input patterns. OR is the averaged overlap of output patterns.*

#### Summation in Representation Neurons

Each I-neuron fires with a predefined gamma frequency between 90 and 160 Hz (corresponding Interspike intervals (ISIs): 6.3–11.1 ms). Thus the frequencies between I-neurons differ by an integer multiple of 1.1 Hz. It is assumed that activated I-neurons start to fire at the same time shortly before the start of the separation process. Therefore, the first input time of a single I-neuron corresponds to ISI of its spiking frequency. The spatial positions of activated I-neurons define the spatial input overlap and determine the similarity of the input patterns (SI).

Random connectivity implies that R-neurons are activated by different strength and at different times by I-neurons. For the sake of simplicity, synaptic weights from I-neurons to R-neurons are assumed to be equal (at the value of 0.4). The excitatory postsynaptic potentials (EPSPs) are generated by Formula (5) and sub linearly added by Formula (6) in the same branch. EPSPs of all dendritic branches are linearly added by their way to the soma but weakened by a passive decay factor of U_*pass*_ = 0.5. The summation processes of EPSPs plays a role for the separation as they integrate the temporal and spatial input and diversify the point in time at which the somatic spiking threshold is exceeded.

The spikes of I-neurons require a constant time of 1 ms for the propagation to the dendrites of R-neurons. Delays from the branches of R-neurons to the soma depend on their location meaning the spatial distance of the branches from the soma. The delays vary from 2 to 16 ms. Branch 1 spikes are delayed by 2 ms, branch 2 are delayed by 4 ms, and so on. Varying delays modify the time of somatic spikes in relation to the specific combination of activated dendritic branches.

The level of activation of an individual R-neuron depends on the input from combined EPSPs from I-neurons. The input to R-neurons is modified individually by the existing random connections of active I-neurons. The activation of R-neurons is a result of spatial and temporal summation EPSPs. The spatial summation within a single branch is sub-linear and processed by Formula 6. This sub-linear summation allows a larger number of activated I-neurons (3–6) to be used. The more EPSPs and the faster the somatic membrane potential rises, the earlier an action potential is generated by exceeding the spiking threshold.

The different connectivity of R-neurons leads to earlier or later spikes in the individual R-neurons. If the neuron that fires the earliest best represents the input, then the selection of this neuron is not only a temporal but also a spatial characteristic of the output. The temporal separation is supplemented by a spatial separation if several R-neurons with different connections from the input are present in the output level.

#### Subthreshold Membrane Potential Oscillations

The somatic membrane potential of R-neurons is additionally varied by SMOs at theta range (5 Hz with an amplitude of 8 mV). The EPSPs of I-neurons reach R-neurons during the through of theta-SMOs. This is achieved by performing a theta phase reset at 75 ms prior to the start of input. The descending phase of a 5 Hz-SMO begins 50 ms later and the lowest excitability is at the time of 150 ms. The excitability reaches the initial level at 200 ms. Then, the excitability increases for another 50 ms. During the input presentation, the temporal and spatial summation of EPSPs on R-neurons is supplemented by the increasing excitability. Even if the input is small growing excitability will generate a spike at the latest on the peak of SMO. In this way, SMOs increase the range of input power that can create a spike. Due to the combination of increasing excitability and the variety of input power, the times of the first spikes between the through and the peak value of SMO (half theta cycle) are stretched.

#### Winner Takes All-Competition

R-neurons compete with each other if the first spiked R-neuron reduces the membrane potential of others by inhibition. The fastest activated R-neuron (caused by the connectivity of active I-neurons) fires first. The winning R-neuron represents the current input. It can happen that more than one R-neuron wins together.

#### Learning

R-neurons can repeatedly win, if they have additional connections from I-neurons activated by a new input. This reduces the SP. However, this weakening of the SP can be compensated by heterosynaptic long-time depression (hsLTD) as a learning mechanism. HsLTD reduces the synaptic weights of connections when postsynaptic firing is generated by the inputs of other synapses. The reduction of the synaptic weights from inactive input units to a firing R-neuron decreases the probability that another input pattern leads to the firing of same R-neuron, since the later input pattern uses connections from I-neurons with reduced synaptic connections. Therefore, hsLTD works like an “immunization” of the present winner neuron against further gains. In other words, by “immunization” it is meant that the learning effect has reduced the weight of some synapses from previous gains.

#### Synfire Chain

We discriminate between temporal and spatial separation. For temporal separation without additional spatial separation, the question arises as to how the temporal precision of spike times (as generated by R-neurons) can be used for the succeeding processes. In our model an extension from temporal to spatial separation is implemented by the ensemble of E-neurons (Figure 6) as a simple synfire chain. The sequences of ET-neurons connected by feedforward connections spread EPSPs. In addition, each ET-neuron is connected to a single parallel E-neuron. The arriving spikes from the ET-neurons enhance the subthreshold membrane potential of the parallel E-neuron. Thus, an EPSP spreads like a wave from one E-neuron to the next. Since spikes in the ET-neurons are temporally ordered according to their sequence, this ordering is transmitted to E-neurons as traveling subthreshold EPSPs. The input into E-neurons from R-neurons alone as well as the input of ET-neurons only produces subliminal excitation in subthreshold excitation, but the combination of both produces a spike. After generating a spike in R-neurons, a spike is generated in the time-specific E-neuron as it coincides with an EPSP from an ET-neuron. The time course of EPSPs in an E-neuron is simulated by Formula 5. The synaptic weights are conveniently chosen at the value of 5.8 between ET-neurons (to be suprathreshold) and between R-neurons and E-neurons at the value of 2.0. A time-neuron (T-neuron) starts the successive spikes of ET-neurons at the beginning of the input (at 10 ms). Time cells firing at specific moments within a cognitive task or experience have been described by Umbach et al. (2020) found in the hippocampus and entorhinal cortex. As shown in refs. Kitamura et al. (2015) and Salz et al. (2016).

### Simulation Parameters

The goal of the simulation is to show pattern separation in a prototypical way in a small SNN that combines the just described conditions.

The simulation program is written by the author (H.L.) in Python 3.7. Parameters for somatic, dendritic, synaptic, and oscillation properties are shown in Supplementary Data Sheet 1. The simulations are calculated with a time resolution of 1 ms. The values and functions of the simulation are determined as follows:

#### EPSPs

EPSPs (e.g., generated if spikes from I-neurons are propagated to the dendrites of R-neurons) are modeled in the form of an alpha function:


(4) $$\text{f}\,\left(\text{EPSP}\right)=k*w*\delta \,t*g^{-\frac{\delta \,t}{\tau }}$$

*where, k* = *0.8 at dendrites for R-neurons and k* = *5.6 at soma for E- and ET-neurons, g* = *2.7 for E-neurons and 1.35 for R-neurons. The decay time of EPSPs at R-neurons is duplicated from 3.5 to*∼*7 ms for enhancing temporal summation. τ* = *1. w is the initial synaptic weight: w(I-R)* = *2.0. w(ET-ET)* = *5.8. w(R-ET)* = *0.4. w(R-E)* = *w(ET-ET) – w(R-ET)* = *5.4.* δ*t is the time difference.*

The general inhibition after spiking a winning R-neuron globally reduces the membrane-potential of all other R-neurons by program code. For convenience, the reduction in membrane potentials of R-neurons is not realized by an inhibitory interneuron fully connected with all R-neurons but rather by a general downgrade of the membrane potential of all R-neurons by 20 mV after an R-neuron produced a spike.

#### Sublinear Summation at Branches

The height of the *x^th^* summed EPSP is computed by:


(5) EP=xEP*1(1-x-1k)

*where EP*^x^* is the height of x*th *EPSP, EP^1^ is the height of the first EPSP and k* = *12. x varies between 1 and 8 and k* = *12, reducing the amount of the 8*th *EPSP to 40%.*

#### Subthreshold Membrane Potential Oscillations

Somatic subthreshold theta oscillations in R-neurons are explicitly modeled by phase-shifted sine functions f(OP):


(6) f⁢(O⁢P)=h*s⁢i⁢n⁢(0.002*π*f⁢q*(t-j*p⁢h))

*where, h* = *8.0 mV is the oscillation amplitude of R-neurons. Formula (7) holds for somatic oscillations in R-neurons. Fq* = 5 Hz. For R-neurons j*ph remains constantly at 75 ms enabling the arrival of spikes from I during the low period of the SMO. The spikes of single I-neurons start after the first ISI.

#### Heterosynaptic Long-Time Depression

Heterosynaptic long-time depression reduces the synaptic weights of inactive dendritic synapses following a somatic spike. For the sake of simplicity, depression is calculated by a fraction of actual weight:


(7) $$w^{\prime }=w*h\,s$$

*where, w’ is the reduced w; hs* = *0.3 is the reduction factor for w.*

Further parameters for somatic, dendritic, synaptic, and oscillation properties are shown in Supplementary Table 1.

## Results

With SNN-parameters as described above, 20 testing packages are scrolled through. Within the same package, the random connections from I-neurons to the dendrites of R-neurons remain constant, but new packages have new random connections. Each test package displays 20 input peak patterns. In a simulation with 20 packages, 400 time-accurate input-spike trains are therefore presented. For each package, 190 pairs of input pattern can be matched between I- and R-neurons. The resulting SP relates to an average of all 400 input presentations and is based on 3,800 compared patterns. Using the simulation procedure, the probabilistic results of separation are generated because the results are calculated by 20 different randomly selected terms of connection.

Exemplarily dendritic and somatic membrane potential courses and calculated overlap values for pairs of input patterns are shown in Figure 3.

### Temporal or Spatial Separation

As Figure 4 shows, **spatial** separation clearly depends on EF. However, spatial separation effects of SP = 60% are observed even without any expansion and for EF values of less than 1, a partial spatial separation remains. For two times as many R-neurons as I-neurons (*EF* = 2) and the spatial SP is already about 80%. It increases to 90% and more when EF increases to 16.

Large differences between the spatial and temporal measurements of separation (Figure 4) are observed. The **temporal** separation along is always very high (over 90%). It is largely independent of EF. Surprisingly, even a single R-neuron separates the input temporally by about 95%, which means that different inputs (also very similar) produce different times of spikes in a single R-neuron. The repetitions of same input (averagely 5 per package) always result in identical spike times. The average spatial overlap of input patterns of about 60% is reduced to about 6% temporal overlap of output patterns in R-neurons representing SP = 90%.

Since spatial separation has mainly been studied so far, the distinction between temporal and spatial separation opens up new avenues for our understanding of the information processing in the brain. In particular, the high-temporal SP of a single neuron opens up new neuronal processes to represent similar objects as different without learning mechanisms.

Two kinds of connectivity (as described in section “Model”) are used: sparse connectivity (0.75 synapses per I-neuron) reduces the temporal SP compared with dense connectivity (2.25 synapses per I-neuron) whereas the spatial SP remains about the same.

### Key Parameters

The differences between spatial and temporal separation along EF raise the question of key influences on both measurements of separation. **Expansion** does not seem to affect the temporal separation. However, it increases spatial separation: a higher number of R-neurons allows the early activation of a neuron that is better connected than any other for a given input.

We then examined whether **partially random connectivity** from I-neurons to R-neurons is essential for temporal separation. Complete connectivity is implemented by linking the 3 dendritic branches of a single R-neuron through all 8 I-neurons. We then examined whether partially random connectivity from I-neurons to R-neurons is essential for temporal separation. SP is calculated in the same way as previous simulations. Full connectivity as well as incomplete random connectivity result in the same temporal SP = 93%. All identic input patters result in identical output.

In addition, it is sometimes reported that **sparse connectivity** is conducive to the pattern separation. So we simulated our model with sparse connectivity. Sparse connectivity boosts temporal but not spatial SP. However, if “sparseness” is defined as “reducing the fraction of active neurons, that make up a “sparse” population code” (Cayco-Gajic and Silver, 2019), then sparseness is also a very important feature for separation in our model. In particular, backward inhibition significantly reduces activity above the threshold of R-neurons and produces a sparse population code in all simulations of our model.

Our results show that neither EF nor incomplete random, but also not sparse connectivity are key parameters for temporal separation. However, EF and incomplete random connectivity are prerequisites for spatial separation.

To study the influence of input parameters on the summation over time, we independently varied the number of activated I-neurons and their firing frequencies. Their individual SP was measured.

### Separation of Quantities

By increasing the number of activated I-neurons, the time to the first spiking of R-neurons is reduced. The output times, therefore represent the strength of the input in an inverse manner. This relationship is a consequence of the summation of the input spikes and is accompanied by an increase in potential by SMO. However, when the I-neurons fire at different frequencies, this strong relationship is reduced. Therefore we simulated our model with full connectivity and with the same frequencies of 90 Hz for all I-neurons. If the connections between I- and R-neurons were random, the relationship between output times and input strength would also be affected. A high correlation would only be given by a statistical mean of as many output neurons as possible. With full connectivity, different but fixed output times (between 11 and 113 ms) are generated by a single R-neuron. Each number (1–8) of the activated I-neurons produces a different output time. This was also true for the average output times when using 128 R-neurons with random connectivity.

The output times are transferred to the firing of various E-neurons with the help of the synfire chain arrangement. Our model allows **counting the number of activated I-neurons** by temporal separation. Each number of activated I-neurons is represented by a specific E-neuron.

### Separation of Frequencies

By increasing the frequency of activated I-neurons, the time to the first spiking of R-neurons is shortened. The output times should therefore correspond to the input frequency. To check this, all I-neurons fire at the same frequency and this frequency is systematically varied. We simulated our model with full connectivity and with frequencies of I-neurons between 50 and 150 Hz with an interval of 10 Hz. In this arrangement, different output times (between 23 and 89 ms) are calculated from a single R-neuron in relation to the spiking frequencies of three I-neurons. Using the synfire chain arrangement, different frequencies of I-neurons lead to the spiking of different E-neurons as the output. The input frequency is represented by specific E-neurons. Our model allows the **representation of frequencies** by temporal separation.

### Separation of Objects

In a further step, an attempt is made to simulate a simple visual object as input using 8 I-neurons that fire at different frequencies. With this we want to find out whether our model also represents objects separately with the input parameter for objects used there. We assume that different properties of environmental objects are represented by the activities of a few I-neurons. As the properties of simplified objects, 3 possible shapes, 3 possible colors, and 2 possible sizes are used. Each object is defined by 3 properties, one for shape, one for color, and one for size. The activation of I-neurons 1, 2, or 3 represents 3 possible forms of the object, the activation of neuron 4, 5, or 6 represents 3 possible colors, and the activation of neuron 7 or 8 represents 2 sizes of the object.


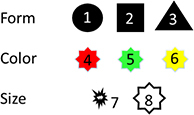


By combining the three properties, our model aims to produce well-separated representations of 16 different objects. Examples of two objects given activated I-neurons as input are shown here:


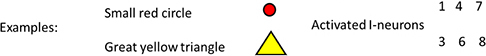


Separation power is tested by presenting all 18 possible objects (plus 2 randomly chosen repeats), each represented by 3 activated I-neurons in our model. The 20 objects are presented in the form of 20 packages, each with a new random connectivity. To reduce the average times in the output from the end of the time domain (caused by activation of only 3 I-neurons) toward its center, the synaptic weights from I-neurons to R-neurons are doubled (from 2 to 4). The SP is measured in two ways: first, as a percentage of correct repetitions of output patterns when the same objects are presented. This criterion by Madar et al. (2019) is named as “input reliability” that is the ability of a neuron to reproduce the same output pattern on the repetitions of input pattern. Second: as a percentage of different output times of the single R-neuron in relation to the number of 18 different input objects. The transformation of spiking times of the R-neurons into spatially different E-neurons is accomplished with the help of a “synfire wave.”

The first result: input reliability is perfect. Approximately 100% of repetitions are represented by equal output times of R-neurons and equal E-neurons.

The second result: the average number of different spike times for 18 different objects is 14. This means that 78% of the different objects are represented by different output times (and subsequently also by different E-neurons). It should be noted that 77% of correct object identification is generated without any learning mechanisms and is based on the spiking times of a single R-neuron. While the spatial overlap of the 18 different input objects is 35% and the spatial overlap of the output patterns of a single R-neuron numbers only to 3.6%. Therefore, similar to previous results the mean temporal SP is 90%.

### Impact of Heterosynaptic Long-Time Depression

The separation effects shown so far do not require any learning mechanisms. They are generated by different excitation with constant synaptic weights. In particular, hsLTD has previously been proposed to facilitate spatial separation effects. This is confirmed in our model (Figure 5). However, this promoting effect of hsLTD is only observed for spatial separation with EF > 1. The temporal separation does not seem to be affected by hsLTD. For EFs of 2 and more, the spatial measurements of SP are over 90%.

### Extension of Temporal to Spatial Separation

Input patterns have produced a time-separated output in R-neurons. In our model the R-neurons temporal output is perfectly converted into a spatial one using “traveling EPSPs” by a synfire chain circuit as described above (as shown in section “Synfire Chain” and Figure 6). Depending on the output time, a different E-Neuron fires. The temporal overlap corresponds exactly to the spatial overlap (Figure 7). There must be as many output neurons available as different input times can occur (about 100 in our simulations).

### Summary of Results

The simulations of our model provide the following results for pattern separation:

• The separation of patterns can be studied in relation to a spatial or temporal aspect of the output. Therefore, the temporal or spatial overlap within the output patterns is measured relative to the spatial overlap within the input patterns generated by a varying number of differently firing neurons. The comparison of the two measurements shows that spatial separation has additional requirements.
• The spatial SP increases with EF and only reaches more than 90% at *EF* = > 8. The temporal measurement even to this SP level can be reached with the help of a single R-neuron. This corresponds to EF of 0.125. In addition, spatial SP requires random connectivity and cannot be produced with full connectivity.
• Sparse connectivity reduces temporal but not spatial SP.
• The number of activated I-neurons and their frequencies affect the output times of R-neurons. These input parameters show up in our model as significant influences for the temporal separation.
• Objects represented by the combination of activated I-neurons can be separated *via* the temporal output of a single neuron. The temporal separation can then be expanded to include a spatial dimension. The temporal overlap of a single R-neuron and the spatial overlap of E-neurons is reduced by about 90% compared with the spatial overlap of I-neurons. Repeated presentations of the same objects always activate the same R-neuron. In total, 18 different objects were used to activate on average 14 different E-neurons. Temporal SP is 90%.
• Heterosynaptic long-time depression does not further improve temporal SP, but improves spatial SP when *EF* > 1
• With the help of a synfire chain, a perfect transformation from a temporal to a spatial separation can be achieved.

The model is reasonably robust to variations in the gamma and theta frequencies used: SMO-theta frequencies of 3 HZ in R-neurons (instead of 5 HZ) and gamma frequencies of the input between 60 and 130 HZ (instead of 90–160 Hz) lead to a reduction in the SP of only about 15%. Both high and low theta oscillations in the human hippocampus have recently been identified by Goyal et al. (2020).

## Discussion

The presented model establishes a new way to study the separation process of patterns by distinguishing temporal and spatial aspects. It turns out that times to the first output spike allow for high separation even in the absence of expansion and even within a single neuron. As shown in Figure 4, the temporal SP comes to more than 90% and is independent of the number of participating R-neurons. This high temporal separability of a single neuron is energy-saving and is able to be present at many circuits where somatic SMOs and summation across dendrites exist. The input-output transformation of our model does not depend on synaptic modification. The different numbers of activated I-neurons, different frequencies of I-neurons, and different delays in propagation of EPSPs from dendritic branches to the soma seem to create the temporal separation force. The separation is processed in conjunction with theta SMOs in the R neurons and a temporal WTA network mechanism. We offer time to the first spike as a crucial aspect of the temporal code relevant to pattern separation. Madar et al. (2019) suggested that separation can be realized by varying the temporal characteristics of output spike trains as spike times, the firing rate, the number of bursts, and the number of spikes in a burst.

Separation processes in the brain are not localized to a specific region (Cayco-Gajic and Silver, 2019). Separation processes presumably take place in all sensory channels and also on the different levels of processing. Our model is simplified to best represent an exemplary separation process with its temporal and spatial aspects.

The following neuronal mechanisms applied by our model are physiologically plausible even if they have not yet been directly investigated in relation to pattern separation in the presented combination.

• **Winner Takes All-mechanism** of a time-based competition, WTA-networks are often used in computational models. The WTA-computation is an intrinsic property of recurrent networks that are abundant in cortex. Several studies have discussed the computing power of spiking WTA networks (Oster et al., 2009). Braganza et al. (2020) outlined a computer model related to backward inhibition in the dentate gyrus and investigated its ability to perform pattern separation. They found a moderate feedback inhibition mediated pattern separation effect during theta-modulated input but a substantial separation, particularly from very similar inputs during gamma oscillations (as used in our model). The impact of backward inhibition for separation is also shown by Wick et al. (2010).
• **Gamma aligned input spike trains** and **theta oscillations of excitability** in output-neurons are the forms of coding that are clearly demonstrated in the hippocampus (Lisman and Jensen, 2013).
• **Theta phase-related input** to R-neurons is essential to realize WTA-competition in our model. The input has to arrive during the low period of the theta SMO. Such phase-related activities often have been found in the hippocampus. For example, pyramidal neurons recorded in the CA1 pyramidal cell layer of awake animals discharge on average in the negative phase of the theta cycle (Buzsaki, 2002). The results of Pernía-Andrade and Jonas (2014) show that the onset of action potentials in hippocampal granule cells is phase-locked to the descending part of the theta and gamma wave. They suggest that action potentials are generated at specific phases of the theta-gamma cycle, and their results are consistent with the idea that a temporal coding scheme is used.
• A **sublinear summation of the synaptic input** to a single branch of R neurons is used. This mechanism is not essential for the proposed separation process, but allows input by a larger number of I-neurons (3–6 in our model) without premature spiking effects. Reports have shown that sublinear summation is a prominent dendritic operation, extending the range of subthreshold input-output transformations conferred by dendrites (Tran-Van-Minh et al., 2015). Findings indicate that a sublinear integration of synaptic inputs is possible in multiple neuron types.
• **Delays of propagation** of EPSP to the soma depending on the spatial distance of synapses at R-neurons. Propagation delays of spikes (arriving at dendritic synapses) on their way to the soma and EPSP attenuation are taken into account. Agmon-Snir and Segev (1993) calculated the time delay and speed of propagation of electrical signals in a passive dendritic tree. The delay contributed by the dendrites in a modeled layer 5 cortical pyramidal cell is l0–17 ms for distal apical arbors and 1.5 ms for the basal dendrites. The net delay is reduced by 6–10 ms for the apical arbors and by 1–1.3 ms for the basal arbor. In our model, implemented delays are again not essential for the separation process even if they can enhance the SP.
• As an only learning effect related to pattern separation, we study the impact of **hsLTD** sometimes considered as relevant for pattern separation (O’Reilly and McClelland, 1994).

In summary, the model presented uses a combination of neural mechanisms to effect a pattern separation. Specific to our model is that the first step of separation is based on spatial and temporal summation effects of membrane potentials in output-neurons. The summation leads to different delays of first spiking.

### Delay Code

Information processing through delay times is not entirely new. Henze et al. (2002) proposed that the information in single granule cells is converted into a time delay code by CA3 pyramidal cells and interneurons. Higher granule cell spike frequencies produce shorter delays. A similar magnitude of activation temporally discharges CA3 targets together, thereby increasing their connectivity to one another. In contrast, our model uses the different distribution of spike times. First spikes in R-neurons are distributed in time and different delays separate the spatially overlapping input patterns. Sufficient summation effects are required to differentiate the input patterns. Temporal summation can be improved by longer EPSPs. Indeed, higher EPSP decay time constants were found in the hippocampus, where separation processes are likely. The decay time constant in dentate gyrus granule cells is about 6 ms and in hippocampal CA3 neurons is 11 ms (Kowalski et al., 2016). We use a decay time constant of 7 ms for R-neurons, for the other neurons in our model we use 3.5 ms.

### Phase Code

According to our model, EPSPs by input patterns arrive the R-neurons during the ascending part of theta SMOs. Due to this temporal position of the input in relation to the theta phase, the lower cumulative values of membrane potential can also become overthreshold due to the increasing oscillation.

Theta oscillations are present in all subregions of the hippocampus and in the granule cells of the dentate gyrus. Increased activity patterns in the target cells of the dentate gyrus or the CA3 region due to input from the entorhinal cortex in the ascending phase of theta waves probably could be indicative of the proposed separation mechanisms. In fact, the overall population of active pyramidal cells fires the most action potentials per theta cycle just after theta cycle bottom (Mizuseky et al., 2009). However, this might be oversimplified to validate our model. Theta oscillations can be generated intrinsically (e.g., Fellous and Sejnowski, 2000 in the CA3 field) or extrinsically by synaptic circuits (e.g., by rhythmic perisomatic inhibition). The physiological mechanisms that evoke theta field potential and the temporal coordination of individual neurons across anatomically sequential subregions through theta oscillations are not yet fully understood. Population activity in hippocampal subregions does not merely reflect their input but also represents the result of autonomous local computation. Mizuseky et al. (2009) found an offset by a half-theta cycle of downstream dendritic excitation of CA3- and dentate gyrus neurons although the peak of population activity in the upstream entorhinal cortex structure corresponded well with the timing of dendritic excitation. Theta dynamic seems to allow for a considerable degree of independence of local circuit computation in the successive stages of the EC-hippocampal system.

### Expansion Recoding

It has always been assumed that expansion recoding plays a key role in pattern separation (Marr, 1971). The expansion can be quantified by the ratio of the size of the input population to the population of the expanded layer. In complete contrast to this, our simulations show that at least high temporal separation can occur through individual neurons and even taking place with a reduction. Spatiotemporal patterns are in a first step separated in the millisecond range of a rising theta wave (∼100 ms). Different spike times replace the commonly used different neurons. Only for the transfer of temporal into spatial separation a moderate expansion is required as a second step, which can be done by different mechanisms, e.g., by synfire waves or by competing WTA with random diluted connectivity. Thus by an expansion factor of 2 a spatial SP of 90% is achieved if supported by hsLTD. That is consistent with Cayco-Gajic and Silver (2019), who suggested that different structural and functional properties can cause pattern separation.

### Implementation in Hippocampus

In the following we try hypothetically to locate our model in the circuits between entorhinal cortex, dentate gyrus, and hippocampal CA3-region, where significant separation processes are thought to be processed. For this purpose, our model is expanded in a modular way: 8 I-neurons together with 16 R-neurons form a unit that is combined to form a larger system of n modules. Every module processes a pool of I-neurons to the R-neurons. R-neurons are trained to be equivalent to the CA3 pyramidal neurons. Their input comes mainly from the perforant path of the entorhinal cortex, from mossy fibers of dentate gyrus, and from CA3 pyramidal neurons themselves by recurrent axons. The entorhinal input is relatively weak rarely exceeding the spiking threshold of pyramidal neurons by itself. The dentate input is arrived by a small number of strong synapses (mossy terminals synapse with 11–15 different CA3 pyramidal cells, Acsády et al., 1998) and does not exceed the spiking threshold on its own. Only the combination of both inputs is able to trigger spikes in CA3 cells. Whether this happen depends on the random connectivity of both inputs. In any case, the random and sparse connectivity from the dentate gyrus limits the field of possible activated CA3 neurons. It determines the effective factor of expansion by the input from the entorhinal cortex. Only in CA3 neurons receiving input from the dentate gyrus, the input from the EC is able to become suprathreshold and to represent the entorhinal input *via* separation. Subsequently, the activation of individual pyramidal cells leads to autoassociative feedback circuits that can generate memories and pattern completion of the input.

Our separation model works within a single theta phase of SMO of about 100 ms. New theta phases produce new separated representations of the next input. Separation can take place sufficiently rapidly to be complete within one theta cycle.

### Increase in Entropy

High temporal SP increase favors the stochastic activity of neurons during information processing, resulting in an increase in entropy, which forms a correlation of perception as hypothesized by Gupta and Bahmer (2019). Note that an increase in SP will lead to a decrease in the probability of joint activity of pairs of neurons if separation is also accompanied by the control of the pairs of neurons by different sets of influences. The decrease in the probability of joint activity will increase the entropy and decrease the mutual information. However, note that the increase in entropy or surprisal information if combined with an increase in mutual information serves as key bases of perception. Thus, the separation allows new time windows for an increase in mutual information given only the presence of specific stimuli. Therefore, the proposed model provides an important mechanism for an increase in entropy during information processing underlying the cognitive functions of the brain.

### Time to Space

We are surprised at the high temporal separation by a single neuron. Nevertheless, temporal differences in the representation patterns after separation are sometimes only one or a few milliseconds. The question arises as to how such small temporal differences can be further processed in a meaningful way. Using synfire chain processes, we make a new proposal how temporal patterns can be extended by neurons to spatial patterns for further processing. To do this, we use systematically altered excitability between neighboring neurons. The temporal separation by single neurons can be perfectly converted back into an additional spatial separation. A very different way of processing small differences in time is expansion, which was seen as central to the separation process. Incomplete random connectivity spreads the times to the first spike across a spatially extended ensemble of neurons. A competing WTA-mechanisms selects the neurons with the shortest delay. There are probably other ways of converting temporal differences into spatial ones.

We hypothesize that additional determinants not considered here, such as the diversity of synaptic weights on dendritic branches can influence the SP. Thus Rolls (2016) noted that pattern separation could be produced by a fully connected competitive net without learning in which the synaptic weights are set to random values.

## Conclusion

A model is presented that defines the pattern separation mainly *via* the temporal dimension of neural activity. Spatially different input patterns lead to an output with different delays due to the spatial and temporal summation processes of membrane potential. SMOs can amplify differences in the delay of output spikes. The output as spikes with different delays can be processed downstream under different conditions. According to our model, the process of spatial separation, which has been mainly investigated up to now, presents itself as a reverse transformation of the time delay into spatial differentiation. The expansion performed by incomplete random connections seems to be responsible for this purpose. By our model, the original separation occurs in individual neurons by summing up the membrane potentials of incoming spikes over time. Other forms of back-transforming the temporal separation by delays into a spatial separation, e.g., by “synfire wave” are also possible. In any case, the time dimension should be more integrated into pattern separation research.

## Data Availability Statement

The original contributions presented in the study are included in the article/Supplementary Material, further inquiries can be directed to the corresponding author/s.

## Author Contributions

HL and DG contributed to conception and design of the study. HL simulated the model and wrote the first draft of the manuscript. DG wrote sections of the manuscript. Both authors contributed to manuscript revision, read and approved the submitted version.

## Conflict of Interest

The authors declare that the research was conducted in the absence of any commercial or financial relationships that could be construed as a potential conflict of interest.

## Publisher’s Note

All claims expressed in this article are solely those of the authors and do not necessarily represent those of their affiliated organizations, or those of the publisher, the editors and the reviewers. Any product that may be evaluated in this article, or claim that may be made by its manufacturer, is not guaranteed or endorsed by the publisher.
