# Supplementary material for: A Model of Pattern Separation by Single Neurons
Source: Front Comput Neurosci. 2022 Apr 29;16:858353. doi: 10.3389/fncom.2022.858353 (PMC9103200; doi:10.3389/fncom.2022.858353)
Supplement: Supplementary file 1 [file Data_Sheet_1.PDF]

```
# IPython 7.8.0 -- An enhanced Interactive Python.
# Spyder 3.3.6

# Hubert Loeffler, Daya Shankar Gupta
# A New Model of Pattern Separation by Single Neurons
# --------------------------------------------------------

import pdb#; pdb.set_trace()
import sys # to exit of the programm
import random # random
import inspect # line number
import math
import numpy as np
import matplotlib.pyplot as plt # plot graphics
import pickle # to store lists
import statistics
import collections
import time

# ==============================================================================
def interrupt():
   programPause = input("Press the <ENTER> key to continue...")

# ==============================================================================
def lineno(): #line number
   return inspect.currentframe().f_back.f_lineno

# ==============================================================================
# Heterosynaptic LTD
def hsLTDsub_neu (T,n,W,Wm,f_hsLTD,nD,C,ST,nI):
```

```
    for b in range(1,nD[n]+1):
        for c in range(0,len(C[n][b])):
            yy = C[n][b][c]
            if max(ST[yy]) == [0] and W[n][b] is not 0:  # no spike at connected neuron, but connections at this branch
                W[n][b][yy-nI-1] = W[n][b][yy-nI-1] * f_hsLTD #!!!!!
    return(T,n,W,Wm,f_hsLTD,nD,C,ST,nI)


# ==============================================================================
# Plot Overlap
def plot_overlap(IO, IO_mean_end, IO_mean_meta,oo,m,first_T):


    for k in range(1, 140):
        IO_mean = []
        if len(IO[k]) > 0:
            for mm in range(0,len(IO[k])):
                IO_mean.append(IO[k][mm])


        if len(IO_mean) > 0:
            IO_mean_end[k].append(statistics.mean(IO_mean)) # Averaged overlap per k for all packages
            #print("Average Output overlap of input overlap of ", k," = ", round(IO_mean_end[k][0],1))


    # Spatiotemporal- and spatial_E-values are not shown
    if (SType[oo] == "spatial" and rate == 1) or SType[oo] == "temporal":
        print("\n","\n")
        print("Package: ", m)
        print(SType[oo])
        print("-----------------------------------")


        Oplot = list(range(0,100))
        plt.xlabel('Input-Overlap [%]')
        plt.ylabel('Output-Overlap [%]')
```

```
        plt.grid(True)

#         for k in range(0, 100):
#             xmin, xmax, ymin, ymax = 0, 100, 0, 100
#             plt.axis([xmin,xmax,ymin,ymax])
#             plt.fill_between(Oplot, 0, 100, color='lightcyan',alpha=0.1)
#             #plt.ylim(-90.0, -30.0)
#
#             if IO_mean_end[k] == []:
#                 q = -10     # should not be plotted
#             else:
#                 q = IO_mean_end[k]
#             plt.plot(k, q, 'bo', markersize=10)
#             plt.plot(k, k, 'k.', markersize=5)
#         plt.show()

    # Appending IO_means over packages
    for i in range(1,141):

        if IO_mean_end[i] == []:
            q = -1     # Should not be plotted
        if IO_mean_end[i] == [0]:
            q = 0
        if IO_mean_end[i] > [0]:
            q = IO_mean_end[i][0]
        IO_mean_meta[i][oo].append(q)

    return()

#""""""""""""""""""""""""""""""""""""""""""""""""""""""""""""""""""""""""""""""""""""""""""""""""""""""""""""""""""""
# Plot Potentials
def plot(b,nT,nN,ST,nI,nE,R,S,M,Eabs,Oabs,nb_R,C,W,nD, a, rate,I, pltE):
```

```
if pltE == 0:
    return()

Tplot = list(range(3,nT))

plt.ylabel('MP [mV]')
plt.xlabel('time [ms]')
plt.grid(True)

print ("a = ",a)

for n in range(1, nN + 1):

    # E-neurons
    if n > nI and n < nI + nE + 1 and pltE == 1:
        b = 0
        print("Extension neuron", n)

        xmin, xmax, ymin, ymax = 3.0, nT, -90.0, -0.0
        plt.axis([xmin,xmax,ymin, ymax])
        plt.fill_between(Tplot, -90.0, -0.0, color='y',alpha = .1)
        plt.ylim(-90.0, -0.0)

        plt.plot(R[n][b],'k--', linewidth = 2.0, markersize=0) # R
        plt.plot(S[n][b], 'k--', linewidth = 2.0, markersize=0)# S
        plt.plot(M[n][b],'m', linewidth = 2.5, markersize=0)   # M
        plt.plot(Eabs[n][b],'r', linewidth = 1.0, markersize=0)# E(abs)
        plt.plot(Oabs[n][b],'b', linewidth = 2.0, markersize=0)# O(abs)

        plt.show()
```

```
# R-neurons
if n > nI + nE and n < nI + nE + nR + 1 and pltE == 2:
    print("\n","Neuron", n, "\n")

    for b in range(0,nb_R+1):
        if b == 0:
            print("Neuron", n, "soma")
            print("Input neurons. ", I[a] )
            print("AP at:", ST[n][0])

            xmin, xmax, ymin, ymax = 3.0, nT, -90.0, 0.0
            plt.axis([xmin,xmax,ymin, ymax])
            plt.fill_between(Tplot, -90.0, 0.0, color='y',alpha = .1)
            plt.ylim(-90.0, 0.0)

            plt.plot(R[n][b],'k--', linewidth = 2.0, markersize=0)
            plt.plot(S[n][b], 'k--', linewidth = 2.0, markersize=0)
            plt.plot(M[n][b],'m', linewidth = 2.5, markersize=0)
            #plt.plot(Eabs[n][b],'r', linewidth = 1.0, markersize=0)
            #plt.plot(Oabs[n][b],'b', linewidth = 2.0, markersize=0)

            plt.show()

        else:

            print("Neuron", n, "branch", b)
            print("Dendritic connections from neurons:", C[n][b])
            print("Activated: ", I[a], " - Therefore:")

            if C[n][b] is not 0:

                if 1 in C[n][b] and 1 in I[a]:
```

```
        print("Input times[1]:",ST[1][0][:8],"...")
    if 2 in C[n][b] and 2 in I[a]:
        print("Input times[2]:",ST[2][0][:8],"...")
    if 3 in C[n][b] and 3 in I[a]:
        print("Input times[3]:",ST[3][0][:8],"...")
    if 4 in C[n][b] and 4 in I[a]:
        print("Input times[4]:",ST[4][0][:8],"...")
    if 5 in C[n][b] and 5 in I[a]:
        print("Input times[5]:",ST[5][0][:8],"...")
    if 6 in C[n][b] and 6 in I[a]:
        print("Input times[6]:",ST[6][0][:8],"...")
    if 7 in C[n][b] and 7 in I[a]:
        print("Input times[7]:",ST[7][0][:8],"...")
    if 8 in C[n][b] and 8 in I[a]:
        print("Input times[8]:",ST[8][0][:8],"...")

# Coincidences of E-spike times with connections
Coin1 = 0
Czero = 0
for j in range(1,nI+1):
    if max(ST[j][0]) > 0:
        Czero = Czero + len(ST[j][0])
if rate == 0:
    if C[n][b] is not 0:
        for ii in range(nI + 1,nE + nI + 1):
            if len(ST[ii][0]) > 1 and ii in C[n][b]:
                Coin1 = Coin1 + 1
    print("Coincident_E-bursts:", Coin1, " for ", Czero , " input times" )

# Print ("Weights from E-neurons to neuron", "n=", n, W[n])
# Print("Syn.Weights", W[n][b])
# Print("Delay:")
```

```
        print("Number of dendritic branches: ", nD[n])

        plt.ylabel('MP [mV]')
        plt.xlabel('time [ms]')
        plt.grid(True)

        xmin, xmax, ymin, ymax = 3.0, nT, -90.0, -0.0
        plt.axis([xmin, xmax, ymin, ymax])
        plt.fill_between(Tplot, -90.0, -0.0, color='y',alpha = .1)
        plt.ylim(-90.0, 0.0)

        #plt.plot(S[n][b], 'k--', linewidth = 2.0, markersize=0)
        plt.plot(R[n][b], 'k--',   linewidth = 2.0, markersize=0)
        plt.plot(Eabs[n][b],'g', linewidth = 1.0, markersize=0)
        plt.plot(M[n][b],'m', linewidth = 2.0, markersize=0)
        #plt.plot(Oabs[n][b],'g--',  linewidth = 2.0, markersize=0)

        plt.show()

# ET-neurons
if n > nI + nE + nR and pltE == 3:
  b = 0
  print("ET - neuron", n, " of ",nN)
  xmin, xmax, ymin, ymax = 3.0, nT, -90.0, 0.0
  plt.axis([xmin,xmax,ymin, ymax])
  plt.fill_between(Tplot, -90.0, 0.0, color='y',alpha = .1)
  plt.ylim(-90.0, 0.0)

  plt.plot(R[n][b],'k--', linewidth = 2.0, markersize=0)
  plt.plot(S[n][b], 'k--', linewidth = 2.0, markersize=0)
  plt.plot(M[n][b],'m', linewidth = 2.5, markersize=0)
```

```
        #plt.plot(Eabs[n][b],'r', linewidth = 1.0, markersize=0)
        plt.plot(Oabs[n][b],'b', linewidth = 2.0, markersize=0)

        plt.show()

    return ()

# ==============================================================================
def variables_begin(nT,nN,nI,nb_E,nb_R,FqR,\
            d_lowTh,k_EP_som,k_EP_dend,Upass,\
            abs_Ref,abs_Ref_d,nC_from,nC_to,nST_from,nST_to):

    print("\n")
    print("A Model of Separation Combining Time and Space")
    print("Hubert Löffler","\n")
    print("SIMULATION PARAMETERS:","\n")
    if rate == 2:
        print("OBJECT-condition")
    print ("nT:        ", nT)
    print("nN:        " ,nN)
    print ("nI:         ", nI)
    print ("nE:        ", nE,"       nb_E:      ",nb_E)
    print ("nR:         ", nR,"       nb_R:      ",nb_R)
    print("Fq_R:        ", "{0:.0f}".format(FqR), "       Ph_R:     ", Ph_R, "      h_R:    ", h_O_R)
    print("k_EP_som:     ",k_EP_som, "      k_EP_dend: ",k_EP_dend)
    print("Upass:       ",Upass,    "      G1_E:      ", G1_E,"    G1_R:   ", G1_R )
    print("w_IE:        ", wI,      "      w_ER:      ",wO )
    print("nSyn_from:    ",nC_from, "       nSyn_to:   ",nC_to)
    print("nD_from:      ",nD_from, "       nD_to:     ",nD_to)
    print("nST_from:     ",nST_from, "       nST_to:    ",nST_to)
    print("hsLTD:       ", hsLTD, "      f_hsLTD:   ", f_hsLTD)
    print("delay:       ", delay)
```

```
    print("Number of patterns:", nA, "   Number of packages:", nM)
    return()

# ==========================================================================
def variables_end_E(a,nI,nE,nN,ST,I,Out_t,rate, f_I):

    # local variables
    # Out, Out_0, Out_1, Out_2, Out_E

    global Out_E

    print("\n")
    print("Results:","\n", "a =",a, "m =", m )

    if a == 1:
        Out_E = [0] * (nA + 1)

    Out[a] = []
    Out_0[a] = []
    Out_1[a] = []
    Out_2[a] = []
    Out_2val[a] = []
    Out_E[a] = [0]

    for n in range(nI + nE + 1, nI + nE + nR + 1):  # R-neurons

        if max(ST[n][0]) > 0:

            z_0 = str(n)
            z_1 = str(n)  + " . " + str(ST[n][0][0])
            z_2 = str(ST[n][0][0])
            z_2val = ST[n][0][0]
```

```
        Out_0[a].append(z_0)
        Out_1[a].append(z_1)
        Out_2[a].append(z_2)
        Out_2val[a].append(z_2val)

if a > 1:
    if Out_E[0] is not 0:
        Out_E = [0] + Out_E

# Rate_spatial(E)
for n in range(nI  + 1, nI + nE + 1):  # only E-neurons
    if max(ST[n][0]) > 0 :
        z = str(n)
        Out_E[a].append(z)
        if 0 in Out_E[a]:
            Out_E[a].remove(0)

print("\n")
if rate < 2:
    print("Input-times: ",  I[a], "\n")

if rate == 2:
    print("Objects by traveling EPSPs:  ","Input-neurons: ",  I[a], "\n")

    if I[a][0] == 1:
        formO = "circle"
    if I[a][0] == 4:
        formO = "square"
    if I[a][0] == 7:
        formO = "triangle"

    if I[a][1] == 2:
```

```
        colourO = "red"
    if I[a][1] == 5:
        colourO = "green"
    if I[a][1] == 8:
        colourO = "yellow"

    if I[a][2] == 3:
        sizeO = "small"
    if I[a][2] == 6:
        sizeO = "great"

    print("OBJECT:  ", sizeO," ",colourO," ",formO, "\n")

print("Output_s:",Out_0[a])
#print("Output_st:",Out_1[a])
print("Output_t:",Out_2[a])
print("Output_E:",Out_E[a], "\n")

# Counting ET-neurons
if nR == 1:
    Outx = []
    if a == nA:
        if 0 in Out_E:
            Out_E.remove(0)
        for x in range(0,len(Out_E)):
            xx = str(Out_E[x])
            Outx.append(xx)

        counter=collections.Counter(Outx)
        nDEN.append(len(counter))
        print("Number of different ET-neurons: ",len(counter))
```

```
#  first spikes
Outx = []
if a == a: #nA:
    for x in range(0,len(Out_2val[a])):
        xx = Out_2val[a][x]
        Outx.append(xx)

    counter=collections.Counter(Outx)
    #nDEN.append(len(counter))

    if first_T == "1":
        print(counter)
        # Plot Input-Outpout Correlation
        for k in range(1, 141):
            # print("k=",k,"oo=",oo)
            # print("IO_mean_meta",IO_mean_meta[k][oo])
#           if len(IO_mean_meta[k][oo]) > 0:
#               # deleting values less than zero
#               e = 0
#               while e < len(IO_mean_meta[k][oo]):
#                   ee = IO_mean_meta[k][oo][e]
#                   if ee < 0:
#                       IO_mean_meta[k][oo].pop(e)
#                       e = e - 1
#                   e = e + 1
#               IO_mean_meta[k][oo][0] = int(statistics.mean(IO_mean_meta[k][oo]))

            Oplot = list(range(1,240))
            plt.ylabel('Number of R-neurons')
            plt.xlabel('Time of first spikes (ms)')
            plt.grid(True)
            plt.fill_between(Oplot, 0, 240, color='greenyellow')
```

```
            xmin, xmax, ymin, ymax = 0, 140,  0, 120
            plt.axis([xmin,xmax,ymin,ymax])
            #plt.plot(k, k, 'k.', markersize=5)
            if counter[k] > 0: # 0 should be plotted
                plt.plot(k, counter[k], 'k*', markersize=15)

            if k >= min(Out_2val[a]) and k <= max(Out_2val[a]):
                plt.plot(k,120,'rs',  markersize=20)

        print("    \n", "Output-Times")
        plt.show()

    return(Out_0)

# ============================================================================
# Simulation
def simulation (a):

    # Order of neurons: I:1-8, E:9-140, R:149-277, ET:278-406

    T = 3
    # inhibition time refresh
    for i in range(nI + nE,nN):
        gI[i] = 0

    while T < nT:
        n = nI + 1 # starting with E-neurons

        # E-neurons are only calculated, if rate-condition =>1 and if nR > 1
        if rate == 1 and nR > 1:
            n = nI + nE + 1 # starting with R-neurons
```

```
  # All neurons
  while n < nN + 1:

    # E-neurons and ET-neurons, but not R-neurons
    if (n > nI and n < nI + nE + 1) or n > nI + nE + nR:

      # Evoked synaptic potentials
      for s in range(0,len(C[n][0])):

        # Number of connections
        x = C[n][0][s]
        if max(ST[x][0]) is not 0:                              # if AP-inputs exist
          for z in range(0,len(ST[x][0])):                      # number of AP-inputs from
connections

            if ST[x][0][z] is not 0 and (max(ST[n][0]) == 0 or ST[x][0][z] < max(ST[n][0])):
              # Actual AP-input > 0 and (no AP in n or AP-input after last Ap in n)
              y = T-ST[x][0][z]
              if y > 0:
                Es[n][0][s][T] =  k_EP_som * W[n][s] * y * G1_E ** (-y)/tau_E
                Esabs[n][0][s][T] = Es[n][0][s][T] + gR
                E[n][0][T] = E[n][0][T] + Es[n][0][s][T]

      # If T > max(ST[n][0]) + 2:                       # if maximal AP in n < T + 2 [absout
refractoriness] (e.g. after bursting)
      Eabs[n][0][T] = E[n][0][T] + R[n][0][T]
      M[n][0][T] =  E[n][0][T] + O[n][0][T] + R[n][0][T]

      # AP-generation in E-neurons or ET-neurons
      if (S[n][0][T] < M[n][0][T]) and T < (nT-3) and max(ST[n][0]) < (T - abs_Ref): # If M exceedes S
and until 3 ms before end  and abs refractoriness

        ST[n][0] = ST[n][0] + [T]
```

```
        M[n][0][T] = 30                              # AP at this time point!

    else:
        if M[n][0][T] is not 30:
            M[n][0][T] = R[n][0][T]

# R neurons
if n > nI + nE and n < nI + nE + nR + 1:

    # Somatic oscillations
    zz = int(str(TOPh[n][0]))
    O[n][0][T] = lowTOH[n][0] * math.sin(0.002*FqR*(T-zz)*math.pi)
    Oabs[n][0][T] = O[n][0][T] + R[n][0][T]

    # Dendritic computations
    for b in range(1,nD[n]+1):
        # Oscillations
        zz = int(str(TOPh[n][b]))
        # Evoked synaptic potentials
        # Connections of b of n
        ss = 1 # for sublinear summation
        for s in range(0,len(C[n][b])):
            x = C[n][b][s]
            if ST[x][0] is not 0:
                # Active input of connection of b of n
                for z in range(0,len(ST[x][0])):
                    # Actual AP-input > 0 and (no AP in n or AP-input after last AP in n
                    if ST[x][0][z] is not 0 and (max(ST[n][b]) == 0 or ST[x][0][z] < max(ST[n][b])):

                        # Selection of neurons with different delays
                        y = T-ST[x][0][z]
                        if y > 0 and W[n][b] is not 0 :
```

```
                    Es[n][b][s][T] =  k_EP_dend * W[n][b][x - nI - 1] * y * G1_R ** (-y/tau_R)

                    # Linear degression
                    E[n][b][T] = E[n][b][T] + Es[n][b][s][T]  * (1-(ss-1)/subSum)

                # Enhance order of input synapses to b of n, after all time points and if input > 0
                if z + 1 == len(ST[x][0]) and ST[x][0][z] > 0:
                    ss = ss + 1
            if T > max(ST[n][b]):
                Eabs[n][b][T] = E[n][b][T] + R[n][b][T]
                M[n][b][T] =  E[n][b][T] + O[n][b][T] + R[n][b][T]

        # AP-generation in R
        # Computation of dendritic input into the soma                                   #
summed Es at somatic level
        for bb in range (1,nD[n]+1):

            # With delay  from branches to soma
            if delay == 1:
                E[n][0][T] = E[n][0][T]  + E[n][bb][T-2*bb]  # * (1-0.7/(1+2.72**(5-bb)))   without
nonlinear summation

            # Without delay from branches to soma
            if delay == 0:
                E[n][0][T] = E[n][0][T]  + E[n][bb][T]  # * (1-0.7/(1+2.72**(5-bb)))

        M[n][0][T] = E[n][0][T] * Upass
        M[n][0][T] = M[n][0][T] + O[n][0][T] + R[n][0][T]
        Eabs[n][0][T] = E[n][0][T] + R[n][0][T]

        # General or local somatic inhibition in R
        if T > gI[n] and gI[n] > 0 and n > nI + nE:
```

```
            M[n][0][T] = M[n][0][T] - 30

        if (S[n][0][T] < M[n][0][T]) and n > nI + nE:

            ST[n][0] = ST[n][0] + [T]

            M[n][0][T] = 30

            # Somatic heterosynaptic LTD by AP
            if hsLTD == 1 and n > nI + nE +1:                   # only for representation neurons
                hsLTDsub_neu (T,n,W,Wm,f_hsLTD,nD,C,ST,nI)

            # local or global inhibition
            if local == "0":
                for i in range(nI + nE,nN):
                    gI[i] = T
                #print("global ","n=",n,"T=",T)
                #interrupt()
            else:
                gI[n] = T
                # print("local ","n=",n,"T=",T)
                # interrupt()

            # Eliminate zero in ST
            if ST[n][0].count(0) > 0 and len(ST[n][0]) > 1:
                ST[n][0].remove(0)

    n = n + 1
# end n

T = T + 1
```

```
    # end T

    return()

# ==========================================================================
# MAIN  MAIN   MAIN   MAIN   MAIN   MAIN   MAIN   MAIN   MAIN   MAIN  MAIN   MAIN   MAIN
MAIN   MAIN   MAIN   MAIN   MAIN
def main():

    global TOPh,O,Oabs,C,ST,E,R,Eabs,M,S,lowTOH,FqR,nD,W,Wm,Es,Esabs

    a = 1

    FqR = 1000/d_lowTh                    # oscillation frequency in R

    I = [0] *(int(nA)+1)                  # input spike trains

    Out_t = []
    Out = [0]*(int(nA)+1)                 # output spike trains
    OverI = [0]*(int(nA)+1)               # separation% input
    OverO = [0]*(int(nA)+1)               # separation % Output

    for i in range(0,int(nA)+1):
       Out[i]=[0]
       OverI[i]=[0]*(int(nA)+1)
       OverO[i]=[0]*(int(nA)+1)

    C = [0]*(nN+1)
    W = [0]*(nN+1)
    Wm = [0]*(nN+1)

    TOPh = [0]*(nN+1)
```

```
lowTOH = [0.0]*(nN+1)


ST = [0]*(nN+1)


nD = [0]*(nN+1)


# CONNECTIONS
for n in range(nI + 1 , nI + nE + 1):
   C[n] = [[]]
   for nn in range(nI + nE + 1 , nI + nE + nR + 1):
      # Fixed connections from R-neurons to E-neurons
      C[n][0].append(nn)
      # Fixed connections from ET-neurons to E-neurons
      C[n][0].append(n + nE + 1)


# Random C and nD at R
for n in range(nI + nE + 1, nI + nE + nR + 1):
   # Number of branches
   nD[n] = random.randint(nD_from, nD_to)


   # Connections,
   C[n] =[0]*(nE+1)
   C[n][0] = [0]


   # Dendritic connections of R
   for b in range(0,nD[n]+1):
      C[n][b] = [0]


      # Random connections to branches
      if b > 0:


         # Rate condition
```

```
        if rate > 0:
            x = random.sample(range(1, nI + 1), random.randint(nC_from,nC_to))
            x.sort()
            for e in range(1,nb_R + 1):
                if e in x:
                    C[n][b] = x
    #end b

# C at ET
for n in range(nI + nE + nR + 1,nN + 1):
    # Only somatic connections
    C[n] = [[n - 1]]

# First ET has connection from I[0]
C[nI + nE + nR + 1][0] = [0]

# WEIGHTS
if Reset_W == 1:
    if Reset_C == 0 or Reset_C == 3:
        with open("nD_PatSep.csv", 'rb') as filehandle:
            nD = pickle.load(filehandle)

    # fixed W,Wm in E
    for n in range(nI + 1, nI + nE + 1): # only every second neuron is connected
        W[n] = [wI, wRT - wI] * (nb_E)    # two weights together transmit threshold S
        Wm[n] = [wI, wRT - wI] * (nb_E)

    # Set W, Wm in ramdomly connected R
    for n in range(nI + nE + 1,nI + nE + nR + 1):
        W[n] = [0]*(nb_R+1)
        Wm[n] = [0]*(nb_R+1)
```

```
        # Dendritc phases and hights of oscillations in R
        for b in range(0,nD[n] + 1):
          # weights in R
          W[n][b] = [0]
          W[n][b] =[(wO)]*nE
          # Maximal synaptic weights of R from E
          Wm[n][b] = [0]
          Wm[n][b] =[(wO + 0.05)]*nE
#         #end b
#       # end n

      #Set W, Wm in ET connected from I and ET-before
      for n in range(nI + nE + nR + 1,nN + 1):
        W[n] = [0]
        Wm[n] = [0]
        W[n][0] = wRT
        Wm[n][0] =wRT
    #--------------------
    # OSCILLATIONS

    # Set Oscilations in ramdomly connected R
    for n in range(nI + nE + 1,nN + 1):
      TOPh[n] = [0]*(nb_R + 1)
      lowTOH[n] = [0]*(nb_R+1)
      # Dendritc phases and hights of oscillations in R
      for b in range(0,nD[n] + 1):
        TOPh[n][b] = Ph_R
        lowTOH[n][b] = h_O_R


      #end b
    # end n
```

```
if int(Reset_C) == 0 or (int(Reset_C) == 3 and m > 1): # load c and nD old
    #  number of branches in R
    with open("nD_PatSep.csv", 'rb') as filehandle:
        nD = pickle.load(filehandle)
    # all connections in R
    with open("C_PatSep.csv", 'rb') as filehandle:
        C = pickle.load(filehandle)

# load w old = learning is present
if int(Reset_W) == 0:
    # w  in R
    with open("W_PatSep.csv", 'rb') as filehandle:
        W = pickle.load(filehandle)

# A
while a <= int(nA):
    Out[a]=[0]
    SToo[a] = []

    # SPIKE TRAINS
    for n in range(nI+1,nN+1):
        # Preparing input spike times
        ST[n]=[0] * (nE+1)
        ST[n][0] = [0]*(nb_R+1)
        # Dendritic spike times
        if n > nI+nE:
            for b in range(1, nb_R+1):
                ST[n][b] = [0]*(nb_R+1)

    # Preparing input spike times
    ST = [0]*(nN+1)
```

```
for n in range(0,nI+1):
    ST[n]=[0]
for n in range(1,nN+1):
    ST[n]= [[0]]*(nE+1)

ST[0][0] = [0]

#Time-neuron for traveling EPSP
ST[0][0] =[5]

if rate > 0:
    ISI = [0] * (nA+1)

    # Spike rates
    ISI[a] = [ISI_1,ISI_2,ISI_3,ISI_4,ISI_5,ISI_6,ISI_7,ISI_8]

# Used input neurons for rate-coding
fq_n = random.sample(range(1, nI+1),random.randint(nST_from,nST_to))

fq_n.sort()

I[a] = fq_n

# RANDOM INPUT FOR OBJECTS
if nR == 1 and rate == 2:

    # Other postion of form, colour and size
    form = [1,4,7]
    colour = [2,5,8]
    size = [3,6]
    I[a] = [random.choice(form),random.choice(colour),random.choice(size)]
```

```
#      manual: n = 3 to 6 random
#       if a == 1:
#           I[a] = random.sample(range(1, nI+1),random.randint(3,3))
#       if a==2:
#           I[a] = random.sample(range(1, nI+1),random.randint(4,4))
#       if a==3:
#           I[a] = random.sample(range(1, nI+1),random.randint(5,5))
#       if a==4:
#           I[a] = random.sample(range(1, nI+1),random.randint(6,6))

        # I is renewed, but if renewing should not happen: old I is set
        if int(Reset_I) == 0 or (int(Reset_I) == 3 and m > 1) : # new input spike trains
            with open("I_PatSep.csv", 'rb') as filehandle:
                I = pickle.load(filehandle)

#       # manuel input
#       if a == 1:
#           I[a] = [1,2,3]
#       if a==2:
#           I[a] = [1,2,6]
#       if a==3:
#           I[a] = [1,5,3]
#       if a==4:
#           I[a] = [1,5,6]
#       if a==5:
#           I[a] = [1,8,3]
#       if a==6:
#           I[a] = [1,8,6]
#       if a==7:
#           I[a] = [4,2,3]
#       if a==8:
#           I[a] = [4,2,6]
```

```
#       if a==9:
#           I[a] = [4,5,3]
#       if a==10:
#           I[a] = [4,5,6]
#       if a == 11:
#           I[a] = [7,2,3]
#       if a==12:
#           I[a] = [7,2,6]
#       if a==13:
#           I[a] = [7,5,3]
#       if a==14:
#           I[a] = [7,5,6]
#       if a==15:
#           I[a] = [7,8,3]
#       if a==16:
#           I[a] = [7,8,6]
#       if a==17:
#           I[a] = [4,8,3]
#       if a==18:
#           I[a] = [4,8,6]
#
#       if a==19:
#           I[a] = I[random.randint(1,16)]
#       if a==20:
#           I[a] = I[random.randint(1,16)]

    # print("I[]", I)


    # Counting input patterns
    if nR == 1:

        Ix = []
```

```
    if a == nA:
        if 0 in I:
            I.remove(0)
        for x in range(0,len(I)):
            xx = str((I[x]))
            Ix.append(xx)

        counter=collections.Counter(Ix)
        # print(counter)
        print("Number of different input patterns: ",len(counter))
        print ("Number of repetitions: ",nA - len(counter))
        nDIP.append(len(counter))
        nRep.append(nA - len(counter))
        I = [0] + I

fq_n = I[a - 1]

if rate > 0: # and int(Reset_I) == 1:
    fq_n = I[a]
    for mm in range(0,len(fq_n)):
        for kk in range(1,24):
            ST[fq_n[mm]][0].append(kk * float(ISI[a][fq_n[mm]-1]))
            # eliminate zero in ST
            if ST[fq_n[mm]][0].count(0) > 0 and len(ST[1][0]) > 1:
                ST[fq_n[mm]][0].remove(0)
            if ST[fq_n[mm]][0].count(0) > 0 and len(ST[1][0]) > 1:
                ST[fq_n[mm]][0].remove(0)
        SToo[a] = SToo[a] + ST[fq_n[mm]][0]

Isum.append(I[a])

if len(I) < nA + 1:
```

```
    print("=",I,"len(I)",len(I))
    print( " ERROR: Loaded input pattern smaller than current ! [", len(I) - 1,"-", nA,"]")
    exit.sys()

# Neuronal parameters
R = [0]*(nN+1)                  # Resting potential
for n in range(nI+1,nN+1):
    R[n] =[0]*(nb_R+1)
    for b in range(0,nb_R + 1):
        R[n][b]=[gR]*nT

S = [0]*(nN+1)                  # Threshold potential
for n in range(nI+1,nN+1):
    S[n]=[0]*(nb_R+1)
    for b in range(0,nb_R + 1):
        if b == 0:
            S[n][b] = [gS]*nT
        else:
            S[n][b] = [dS]*nT
dO = [0,0]                      # Theta oscillation Hight

TOFq = [0]*(nN+1)               # Oscillation frequency
for n in range(nI+nE+1,nN+1):
    TOFq[n] =[FqR]*(nb_R+1)
    for b in range(0,nb_R+1):
        TOFq[n][b]=FqR

# Compute oscillation potential
# O, Oabs, E, Eabs, D, Dabs, Ddabs, DEO, M, Es[], Esabs[]
M = [0]*(nN+1)                  # Membran potential
for n in range(nI+1,nN+1):
    M[n] =  [0]*(nb_R+1)
```

```
    for b in range(0,nb_R+1):
        M[n][b] = [0]*nT
        for t in range(0,nT):
            M[n][b][t] = 0.0

E = [0]*(nN+1)                        # Summed EPSPs
for n in range(nI+1,nN+1):
    E[n] =  [0]*(nb_R+1)
    for b in range(0,nb_R+1):
        E[n][b] = [0]*nT
        for t in range(0,nT):
            E[n][b][t] = 0.0

Eabs = [0]*(nN+1)                     # Summed EPSPs absolute
for n in range(nI+1,nN+1):
    Eabs[n] =  [0]*(nb_R+1)
    for b in range(0,nb_R+1):
        Eabs[n][b] = [0]*nT
        for t in range(0,nT):
            Eabs[n][b][t] = 0.0

O = [0]*(nN+1)                        # Oscillation potential
for n in range(nI+1,nN+1):
    O[n] =  [0]*(nb_R+1)
    for b in range(0,nb_R+1):
        O[n][b] = [0]*nT
        for t in range(0,nT):
            O[n][b][t] = 0.0

Oabs = [0]*(nN+1)                     # Oscillation potential absolute
for n in range(nI+1,nN+1):
    Oabs[n] =  [0]*(nb_R+1)
```

```
    for b in range(0,nb_R+1):
        Oabs[n][b] = [0]*nT
        for t in range(0,nT):
            Oabs[n][b][t] = 0.0

D = [0]*(nN+1)                        # Dendritic spike potential
for n in range(nI+1,nN+1):
    D[n] =  [0]*(nb_R+1)
    for b in range(0,nb_R+1):
        D[n][b] = [0]*nT
        for t in range(0,nT):
            D[n][b][t] = 0.0

Dabs = [0]*(nN+1)                     # Dendritic spike potential at soma absolute
for n in range(nI+1,nN+1):
    Dabs[n] =  [0]*(nb_R+1)
    for b in range(0,nb_R+1):
        Dabs[n][b] = [0]*nT
        for t in range(0,nT):
            Dabs[n][b][t] = gR

Ddabs = [0]*(nN+1)                    # Dendritic spike potential at branch absolute
for n in range(nI+1,nN+1):
    Ddabs[n] =  [0]*(nb_R+1)
    for b in range(0,nb_R+1):
        Ddabs[n][b] = [0]*nT
        for t in range(0,nT):
            Ddabs[n][b][t] = 0.0

DEO = [0]*(nN+1)                      # Branch spike potential
for n in range(nI+1,nN+1):
    DEO[n] =  [0]*(nb_R+1)
```

```
    for b in range(0,nb_R+1):
        DEO[n][b] = [0]*nT
        for t in range(0,nT):
            DEO[n][b][t] = 0.0

Es  = [0]*(nN+1)                       # EPSP per synapse
for n in range(nI+1,nN+1):
    Es[n] = [0]*(nb_R+1)
    for b in range(0,nb_R+1):
        Es[n][b] = [0]*(nb_R+1)
        for s in range(0,nb_R+1):
            Es[n][b][s] = [0]*nT
            for t in range(0,nT):
                Es[n][b][s][t] = 0.0

Esabs  = [0]*(nN+1)                    # EPSP per synapse absolute
for n in range(nI+1,nN+1):
    Esabs[n] = [0]*(nb_R+1)
    for b in range(0,nb_R+1):
        Esabs[n][b] = [0]*nb_R
        for s in range(0,nb_R):
            Esabs[n][b][s] = [0]*nT
            for t in range(0,nT):
                Esabs[n][b][s][t] = gR

#  Parameters for print
if var_begin == 1 and a == 1 and m == 1:

    variables_begin(nT,nN,nI,nb_E,nb_R,FqR,d_lowTh,k_EP_som,k_EP_dend,Upass,\
            abs_Ref,abs_Ref_d,nC_from,nC_to,nST_from,nST_to)

# $$$$$$$$$$$$$$$$$$$$$$$$$$$$$$$$$$$$$$$$$$$$$$$$$$$$$$$$$$$$$$$$$$$$$$$$$
```

```
    simulation (a)
    # $$$$$$$$$$$$$$$$$$$$$$$$$$$$$$$$$$$$$$$$$$$$$$$$$$$$$$$$$$$$$$$$$$$$$


    # plotting
    if pltE > 0:


        plot(b,nT,nN,ST,nI,nE,R,S,M,Eabs,Oabs,nb_R,C,W,nD,a,rate,I,pltE)


    # Results after encoding
    if var_end == '1':


        variables_end_E(a,nI,nE,nN,ST,I,Out_t,rate,f_I)


    a = a + 1


# end a


# Save I
with open("I_PatSep.csv", "wb") as filehandle:
    pickle.dump(I, filehandle)


# Save C
with open("C_PatSep.csv", "wb") as filehandle:
    pickle.dump(C, filehandle)


# Save nD
with open("nD_PatSep.csv", "wb") as filehandle:
    pickle.dump(nD, filehandle)


# Save W
with open("W_PatSep.csv", "wb") as filehandle:
    pickle.dump(W, filehandle)
```

```
    return(SI,SR,SP,n_zero)   # end main


# ------------------------------------------------------------------------------
def sep():


    for oo in range(0,4):
        if oo == 0:
            Out = Out_0
        if oo == 1:
            Out = Out_1
        if oo == 2:
            Out = Out_2
            if len(Out_2[a+1]) > 0:
                Out_meta[oo].append(int(Out_2[a+1][0]))


            #print("len(Out_meta[oo][1:])",len(Out_meta[oo][1:]))
            #print("nM+1",nM + 1)


            if len(Out_meta[oo]) > 0 and m == nM + 1:
                if Out_meta[oo][0] == 0:
                    Out_meta[oo].remove(0)
                if len(Out_meta[oo][1:]) > 0:
                    print("Mean Out_meta[temporal]: ",round(statistics.mean(Out_meta[oo][1:])))
                    print("Mean StandardD[temporal]     : ",round(np.std(Out_meta[oo][1:])))
                    print("Minimum Out_meta[temporal]: ",round(min(Out_meta[oo])))
        if oo == 3:
            Out = Out_E


        OverI = [0]*(int(nA)+1)              # overlap% input
        OverO = [0]*(int(nA)+1)              # overlap % Output
        j_zero = [0]                         # list opf zero_output
```

```
jI_100 = [[0]]                    # list of Input-overlap == 100
for i in range(0,int(nA)+1):
    OverI[i]=[0]*(int(nA)+1)
    OverO[i]=[0]*(int(nA)+1)

IO = [0]*141
OO = [0]*141

for k in range(0,141):
    IO[k]=[]

IO_mean_end = [0] * 141
for k in range(0, 141):
    IO_mean_end[k] = []

with open("I_PatSep.csv", 'rb') as filehandle:
    I = pickle.load(filehandle)    # load input patterns

nI_0 = 0        # number of Input Overlaps with 0%
nI_100 = 0      # number Input Oveerlaps with 100%
nI_mix = 0      # number Input Oveerlaps between 0 and  100%  (mixed)

nO_0 = 0        # number of Output overlaps with 0%
nO_100 = 0      # number of Output overlaps with 100%
nO_mix = 0      # number ov Output voerlaps between 0 and 100% (mixed)

OverI_mix = 0    # summed value of mixed Input overlap

OverO_100 = 0    # summed value of 100% Input overlap
OverO_mix = 0    # Outpur Overlap if Input Overlap = between 0 and 100 (mixed)

n_zero = 0      # number of Output == 0
```

```
z = 0           # number of equal items in Inputpairs

zz = 0          # number of all items in Inputpairs / 2


# Because counter zero was deleted

if Out[0] is not 0:

   Out = [0] + Out


# Zero outputs

for i in range(1,nA+1):

   if Out[i] == []:

      n_zero = n_zero + 1                          # number of zero_output

      j_zero.append(i)

n_zero = len(j_zero)-1


for i in range(1,nA):

   for j in range(i+1,nA+1):


      if rate == 1:

         Isum[i] = I[i]

         Isum[j] = I[j]


      # Input overlap with equal input times

      yy = 0

      z = len(Isum[i]) + len(Isum[j])                # nIP1 + nIP2 (denominator)

      zz = 2*len(set(Isum[i]) & set(Isum[j]))         # niP12  (numerator)


      # Overlap of input pairs:

      OverI[i][j] = int(100*zz/z)


      # Equal input pairs

      if OverI[i][j] == 100:

         jI_100.append([i,j])
```

```
        # Overlap of representation pairs:
        if len(Out[i]) > 0 and len(Out[j]) > 0:
            yyy = list(set(Out[i]) & set(Out[j]))
            y = len(Out[i]) + len(Out[j])                 # nOP1 + nOP2  (denominator)

            for x in range(0,len(yyy)):
                yy = yy + Out[i].count(yyy[x]) + Out[j].count(yyy[x])

            if yy == 0:
                yy = 2*len(set(Out[i]) & set(Out[j]))      # nOP12  (numerator)

            if y > 0:
                OverO[i][j] = int(100*yy/y)               # % output Overlap
    # end for j
# end for i

#print("i=",i,"j=",j,"Input_overlap = ", OverI[i][j],"     Output-overlap = ", OverO[i][j])

n_Over = 0            # number of overlap-comparisons
Dif_Over = 0           # Difference of Overlaps between input and output
nIO_right = 0          # right input Output of 100% overlaps
OverI_p = 0            # Summed overlap I
OverO_p = 0             # summed Overlap O

for i in range(1,nA):
    for j in range(i+1,nA+1):
        # Without equal input pairs and without zero output
        # j_zero: list of zero_output
        # jI_100: list of (Input-overlap == 100)
        if (i not in j_zero and j not in j_zero) and ([i,j] not in jI_100):
```

```
            Dif_Over = Dif_Over + (OverI[i][j] - OverO[i][j])
            n_Over = n_Over + 1

            # summed OverI, summed OverO
            OverI_p = OverI_p + OverI[i][j]
            OverO_p = OverO_p + OverO[i][j]

            if OverI[i][j] < 100 and len(Out[i]) > 0 and len(Out[j]) > 0:
                IO[OverI[i][j]].append(OverO[i][j])

        # equal input pairs
        if OverI[i][j] == 100:
            nI_100 = nI_100 + 1
            if OverO[i][j] == 100:
                nIO_right = nIO_right + 1

        if OverO[i][j] == 100:
            nO_100 = nO_100 + 1

plot_overlap(IO,IO_mean_end,IO_mean_meta,oo,m, first_T)

if nA > 1 and ((oo == 0 and rate == 1) or oo > 1): # only spatial (if rate == 1) and temporal
    print("Equal input pairs ", nI_100, "     right:",nIO_right)
    print("Nr of zero Output: ",n_zero) #, "[",j_zero,"]")

    if n_Over > 0:
        OverI_p = round(OverI_p/n_Over,1)
        OverO_p = round(OverO_p/n_Over,1)

    if OverI_p > 0:
        SP =  round(100*(1 - OverO_p/OverI_p))
        #print("OverO_p",OverO_p,"OverI_p",OverI_p)
```

```
            #if oo == 0 and rate == 1:
            print("SP = ", SP, "%", "\n")
        else:
            print("Overlap of all input patterns == 0!")

        print("-----------------------------------------------------")
        SI_meta[oo].append(OverI_p)
        SR_meta[oo].append(OverO_p)
        if OverI_p > 0:
            SP_meta[oo].append(SP)

        n_zero_meta[oo].append(n_zero)
        nI_100_meta[oo].append(nI_100)
        nO_100_meta[oo].append(nO_100)
        nIO_right_meta[oo].append(nIO_right)

    # enf for oo

    return()

#  META  META  META
#-------------------------------------------------------


    # A New Model of Pattern Separation by Single Neurons
    # Hubert Loeffler, Daya Shankar Gupta

    # nA: number of spike trains to calculate
    # Reset_W: 1: no reset; 0: resetting
    # Reset_I: 1: no reset; 0: resetting
    # Reset_C: 1: no reset; 0: resetting
    # R + number: Continuation of simulation without resetting weights, Connections, input patterns
```

R + numer + c: Continuation of simulation without resetting weights and Connections, but with new input patterns

a: number of spike trains to calculate (intern variable)

dOA: learning by amplification of oscillation

hsLTD: Heterosynaptic LTD by somatic spikes

pltE: plot variables during encoding

var_begin: print simulation parameters before simulation

f_hsLTD: learning factor for hsLTD

ProLTP: lesrning factor for dendritic LTP

nT: final time point of simulation (ms)

nN: number of simulated neurons (1,2,3)

nI: number of input neurons (1,2)

nb_E: number of branches in E

nb_R: number of branches in R

k_EP_dend: k for EPSP at branches in f(EPSP)

k_EP_som: k for EPSP at soma in f(EPSP)

G1 constant related to EPSP

Upass: passive decay of branch potential to soma

abs_Ref: absolute refractoryness of somatic sikes

abs_Ref_d: absolute refractoriness of dendritic spikes

dNMDA: duration of NMDA-spike

gR: general resting potential at all neurons at soma and at all branches

gS: general somatic spiking threshold

dS: general dendritic spiking threshold

FqE: oscillation frequency in E

FqR: oscillation frequency in R

C: connections

W: synaptic weight for EPSP caused by ariving AP (somatic and dendritic)

Wm: maximal weightof dendritic synapses

ST: spike times (somatic and dendritic)

R: actual resting potential

S: actual spiking threshold

O: oscillation potential (0,13): 0 = at soma, 1-12 = at dendritic branches

TOFq: theta-oscillation frequency (somatic and dendritic)

TOPh: theta-oscillation phase (somatic and dendritic)

M: actual membran potential

E: actual evoked potetial: 0 = soma; 1-12 = dendritic

Eabs: absolute E

O: actual SMO potential

Oabs: absolute O

D: dendritic spike potential

Dabs: absolute D

Ddabs: absolute dendritic spike potential

DEO: branch spike potential [= D+E+O]

Es: sum of all EPSPs at the branch)

Ref: function for relative somatic refractorines

n: neurons (0,nN); [1 = neuron I; 2 = neuron A; 3 = neuron E]

b: dendritic branches (0,13) of neuron 3

Delay of propagation from E-neurons to R-neurons

rate: Condition of input (rate, rate+object, point)

SP_mean: Separation Power mean of packages

SI_mean: Separation Overlap in I-neurons mean of packages

SR_mean: Separation Overlap in R-neurons mean of packages

SP_s_mean: Separation Standard deviatipon

Out_meta: Output(oo) mean of packages

n_zero_meta: Number of zero- output mean over packeages

nI_100_meta: Number of eqaul Input patterns mean over packages

nO_100_meta: Number of eqaul Ouput patterns mean over packages

nIO_right_meta: Number of right equal Ouput patterns mean over packages

IO_mean_meta:

Out_0: Spatial Output (R-neurons)

Out_1 Spatiotemporal Output (R-neurons)

```
    # Out_2 Temporal Output (R-neurons)
    # Out_3 Spatial Output (E-neurons)

    # ISI_1 to ISI_8: Inter Spike Inerval of I1 to I8
    # Isum: Input-spike times by frequencies

    # oo: Output overlap (0,1,2,3 - spatial, temporal, spatiotemporal, spatial_E)

    # nDIP: Number of diffeerent Input Pattern (Object)
    # nRep: Number of Repetitions (Object)
    # nDEN: Number of different E-neurons (Object)

    # m: Numerator packages
    # a: Numerator Input patterns

    # first_T:  plotting output times ???
    # nST_from:
    # nST_to:

global subSumn, DIP, nRep, nDEN, Dabs, SType, local

        #main
global Out_t,I,Isum,m,nN,nT,nI,nE,nR,nb_R,nb_E,gR,gS,dS,d_lowTh,h_O_R,Ph_R,\
        k_EP_som,k_EP_dend,G1_E,G1_R,Upass,abs_Ref,abs_Ref_d,\
        wI,wO,hsLTD,f_hsLTD,delay, Out_0,Out_1,Out_2,Out_3,\
        nC_from,nC_to,nD_from,nD_to,nST_from,nST_to,\
        ISI_1,ISI_2,ISI_3,ISI_4,ISI_5,ISI_6,ISI_7,ISI_8,\
        Reset_C,Reset_I,Reset_W,nA,nM,\
        pltE, rate,var_begin, var_end, f_I, tau_E, tau_R

        #sep
global Out_meta,SI_meta,SR_meta,SP_meta,n_zero,n_zero_meta, nA, nI_100_meta,\
```

```
        nO_100_meta, SToo

t1 = time.time()
SI = 0
SR = 0
SP = 0
n_zero = 0

SType =["spatial", "spatiotemporal","temporal", "spatial_E"]
a = 0

Out_t = []

SI_mean = [0,0,0,0]
SR_mean = [0,0,0,0]
SP_mean = [0,0,0,0]
SP_s_mean = [0,0,0,0]
SP_s = [0,0,0,0]

SI_meta = [[0],[0],[0],[0]]
SR_meta = [[0],[0],[0],[0]]
SP_meta = [[0],[0],[0],[0]]
Out_meta = [[0],[0],[0],[0]]

n_zero_meta = [[0],[0],[0],[0]]
nI_100_meta = [[0],[0],[0],[0]]
nO_100_meta = [[0],[0],[0],[0]]
nIO_right_meta = [[0],[0],[0],[0]]
IO_mean_meta = [0] * 141
for k in range(0, 141):
   IO_mean_meta[k] = [[],[],[],[]]
```

```
# Read from csv

# d = open("Spyder_GLOBAL.csv")

#d = open("C:/Users/Hubert/Desktop/Spyder_GLOBAL.csv")

d=open("//Pc/c/Users/Hubert/Documents/__NN/__Spyder/_Spyder_temporal pattern
separation/GLOBAL/Spyder_GLOBAL.csv")

all = d.read()

d.close()


# Read Itemlist

linelist = all.split(chr(10))

itemlist = linelist[1].split(";")


# neurons

nT = int(itemlist[1])              # number of processed time

nI = int(itemlist[2])              # number of Input-neurons

nE = int(itemlist[3])              # number of Extension-neurons

nET = nE                           # number of ET-neurons

nR = int(itemlist[4])              # number of Representation-neurons

nb_R = int(itemlist[5])            # number of potential connections of branches in R from E

nb_E = int(itemlist[6])            # number of branches in E

gR = float(itemlist[7])            # general resting potential at all neurons at soma and at all branches

gS = float(itemlist[8])            # general somatic spiking threshold

dS = float(itemlist[9])            # general dendritic spiking threshold


# oscillations

itemlist = linelist[3].split(";")

d_lowTh = int(itemlist[1])         # duration of somatic Theta-oscillastion phase of Output neurons

Ph_R = int(itemlist[2])            # first somatic Theta-oscillastion phase

h_O_R = float(itemlist[3])         # generallow Theta oscillation hight


# functions

itemlist = linelist[5].split(";")
```

```
k_EP_som = float(itemlist[1])       # constant for somatic EPSP
k_EP_dend = float(itemlist[2])      # constant for dendritic EPSP
G1_E = float(itemlist[3])          # constant related to EPSP in E
G1_R = float(itemlist[4])          # constant related to EPSP in R
Upass = float(itemlist[5])         # passive EPSP decay to soma
abs_Ref = int(itemlist[6])         # absolute refractoryness of somatic spikes
abs_Ref_d = int(itemlist[7])       # absolut refractoryness of dendritic spikes
tau_E = int(itemlist[8])           # EPSP_time contant in E
tau_R = int(itemlist[9])           # EPSP time constant in R

# weights
itemlist = linelist[7].split(";")
wI = float(itemlist[1])            # synaptic weights of E from I or from R
wO = float(itemlist[2])            # synaptic weight of R  from E
wRT = float(itemlist[3])           # synaptic weight of RT from I and from RT-before
subSum = float(itemlist[4])        # subliminar summation at R
hsLTD = float(itemlist[5])         # hsLTD on/off
f_hsLTD = float(itemlist[6])       # factor oh hsLTD
delay = int(itemlist[7])           # delay

# net
itemlist = linelist[11].split(";")
nC_from = int(itemlist[1])         # number of connections from
nC_to = int(itemlist[2])           # number of connections to
if nC_to > nb_R:
   print("nb_R to less! nb_R enhanced to ", nC_to)
   nb_R = nC_to + 1
nD_from = int(itemlist[3])         # number of dendrites of Representation neurons from
nD_to = int(itemlist[4])           # number of dendrites of Representation neurons to
nST_from = int(itemlist[5])        # number of input spike times (from)
nST_to = int(itemlist[6])          # number of input spike times (to)
```

```
if nST_to > nI:
    print("nI to less! n_I enhanced to ", nST_to)
    nI = nST_to

nHz = int(itemlist[7])              # Frequency I-neuron

# input
itemlist = linelist[13].split(";")
Reset_C = int(itemlist[1])          # New connections
Reset_I = int(itemlist[2])          # New input I
Reset_W = int(itemlist[3])          # New weights
nA = int(itemlist[4])               # number of input spike trains
nM = int(itemlist[5])               # number of packages
rate = int(itemlist[6])             # input condition: 0:  1:  2: object condition

if rate == 2 and nR > 1:
    print("\n","Only 1 R-neuron allowed for object condition: nR is reduced to 1")
    nR = 1
    interrupt()

itemlist = linelist[nHz + 10].split(";")
ISI_1 = str(itemlist[8])            # ISI of I1
ISI_2 = str(itemlist[9])            # ISI of I2
ISI_3 = str(itemlist[10])           # ISI of I3
ISI_4 = str(itemlist[11])           # ISI of I4
ISI_5 = str(itemlist[12])           # ISI of I5
ISI_6 = str(itemlist[13])           # ISI of I6
ISI_7 = str(itemlist[14])           # ISI of I7
ISI_8 = str(itemlist[15])           # ISI of I8

#output
itemlist = linelist[15].split(";")
```

```
pltE = int(itemlist[1])             # plot: 1 == E-neruons, 2== R-neurons, 3 == TE-neurons
var_begin = int(itemlist[2])
var_end = (itemlist[3])
first_T = (itemlist[4])
local = (itemlist[5])

nN = nI + nE + nR + nET

gI = [0]*(nN+1) # local inhibition is set zero

Out_0 = [0]*(nA+1)
Out_1 = [0]*(nA+1)
Out_2 = [0]*(nA+1)
Out_2val = [0]*(nA+1)
Out_3 = [0]*(nA+1)

nDIP = []
nRep = []
nDEN = []

f_I = [0]*(nA+1)
SToo = [0]*(nA+1)

for m in range(1,nM+1):

   I = []
   Isum = [0]

   # Load C
   if Reset_C == 0:
      with open("C_PatSep.csv", "rb") as filehandle:
         C = pickle.load(filehandle)
```

```
    main()

    # load I
    with open("I_PatSep.csv", "rb") as filehandle:
        I = pickle.load(filehandle)

    sep()

    # Save I
    with open("I_PatSep.csv", "wb") as filehandle:
        pickle.dump(I, filehandle)

# end for m

print("\n", "Summarized output of all packages")

if m > 1:
    if nA > 1:
        for oo in range(0,4):

            SI_meta[oo].remove(0)
            SR_meta[oo].remove(0)
            SP_meta[oo].remove(0)
            if 0 in n_zero_meta[oo]:
                n_zero_meta[oo].remove(0)
            nI_100_meta[oo].remove(0)
            nO_100_meta[oo].remove(0)
            nIO_right_meta[oo].remove(0)
            if len(SI_meta[oo])> 0:
                SI_mean[oo] = statistics.mean(SI_meta[oo])
            if len(SR_meta[oo])> 0:
```

```
        SR_mean[oo] = statistics.mean(SR_meta[oo])
    if len(SP_meta[oo]) > 0:
        SP_mean[oo] = statistics.mean(SP_meta[oo])
        SP_s[oo] = statistics.stdev(SP_meta[oo])
    if len(nI_100_meta[oo])> 0:
        nI_100_mean = statistics.mean(nI_100_meta[oo])

    # nO_100_mean[oo] = statistics.mean(nO_100_meta[oo])
    # print("SI:", SI_meta,"   SI_mean:", round(SI_mean,1))
    # print("SR:", SR_meta,"          SR_mean:", round(SR_mean,1))
    # print("SP_meta:", SP_meta,"                          SP_mean:", round(SP_mean,1))

    if (oo == 0 and rate == 1) or oo > 1: # only temporal (rate == 2) or spatial and temporal (rate
==1)
        print("\n")
        print("SType: ", SType[oo])
        print("------------------------------------")
        print("All equal input pairs: ",sum(nI_100_meta[oo]))
        print("Right input pairs: ",sum(nIO_right_meta[oo]))

        if len(n_zero_meta[oo]) > 0:
            print("Average zero_output: ", round(statistics.mean(n_zero_meta[oo]),1))
        else:
            print("Average zero_output:  = 0")

        # Plot Input-Outpout Correlation
        for k in range(1, 141):
            if max(IO_mean_meta[k][oo]) > 0:
                # deleting values less than zero
                e = 0
                while e < len(IO_mean_meta[k][oo]):
                    ee = IO_mean_meta[k][oo][e]
```

```
            if ee < 0:
                IO_mean_meta[k][oo].pop(e)
                e = e - 1
            e = e + 1
        IO_mean_meta[k][oo][0] = int(statistics.mean(IO_mean_meta[k][oo]))

    Oplot = list(range(1,101))
    plt.xlabel('Input-Overlap [%]')
    plt.ylabel('Output-Overlap [%]')
    plt.grid(True)
    plt.fill_between(Oplot, 0, 100, color='lightcyan',alpha=0.1)
    xmin, xmax, ymin, ymax = 0, 100, 0, 100
    plt.axis([xmin,xmax,ymin,ymax])
    plt.plot(k, k, 'k.', markersize=5)
    if IO_mean_meta[k][oo][0] >= 0: # 0 should be plotted
        plt.plot(k, IO_mean_meta[k][oo][0], 'ro',markersize=10)

print("    \n", "Iput-Output-Matrix of all packages")

plt.show()

print("mean_OI_",SType[oo],":     ", round(SI_mean[oo],1))
print("mean_OR_",SType[oo],":      ", round(SR_mean[oo],1))
print("mean_SP_",SType[oo],":     ", round(SP_mean[oo],1))
print("standard deviation_SP:   ", round(SP_s[oo],1))

if oo == 3 and nR == 1:
    print("\n")
    print("Separation in E:" )
    if len(nRep) > 0:
        print("Mean Repetitions: ", round(statistics.mean(nRep),1))
    if len(nDEN) > 0:
```

```
            print("nDEN",nDEN)

            print("Mean nE: ", round(statistics.mean(nDEN),1))

          print("---")

          if len(nDIP) > 0:

            print("Mean nObj: ", round(statistics.mean(nDIP),1))

          if len(nDEN) > 0 and len(nDIP) > 0:

            print("Percent Identification of Objects: ",
round(100*(statistics.mean(nDEN)/statistics.mean(nDIP)),1),"%")

      # end if


    #end for oo

  else:


    print("Only one input pattern")


# end meta
```
